# Condition-Specific Protocols Used in the Emergency Department Observation Unit: A Scoping Review

**DOI:** 10.1016/j.acepjo.2026.100425

**Published:** 2026-05-27

**Authors:** Kian D. Samadian, Andrew D. Luo, Malia Pollock, Henry Chen, Joshua J. Baugh, Carl T. Berdahl, Melissa A. Meeker, David A. Meguerdichian, Christopher W. Baugh

**Affiliations:** 1 Department of Emergency Medicine, Massachusetts General Hospital, Harvard Medical School, Boston, Massachusetts, USA; 2 Brigham and Women's Hospital, Department of Emergency Medicine, Harvard Medical School, Boston, Massachusetts, USA; 3 Middlebury College, Middlebury, Vermont, USA; 4 Harvard University, Boston, Massachusetts, USA; 5 Departments of Medicine and Emergency Medicine, Cedars-Sinai Medical Center, Los Angeles, California, USA; 6 Department of Emergency Medicine, Beth Israel Lahey Hospital and Medical Center, Burlington, Massachusetts, USA

**Keywords:** observation unit, emergency department, clinical protocols, accelerated diagnostic pathways, scoping review, clinical observation units

## Abstract

**Background:**

Emergency Department Observation Units (EDOUs) provide time-limited care for patients not clearly requiring inpatient admission. Despite widespread adoption, evidence describing condition-specific EDOU protocols remains fragmented. We conducted a scoping review to map the clinical conditions, characteristics, and outcomes of published EDOU protocols.

**Methods:**

This review follows the Preferred Reporting Items for Systematic Reviews and Meta-Analyses Extension for Scoping Reviews (PRISMA-ScR) guidelines. PubMed was searched for articles published between January 1, 2000, and June 1, 2025. Eligible publications reported condition-specific EDOU clinical care pathways for adult populations. Articles were screened and extracted by 2 reviewers in Covidence. Findings were summarized using descriptive statistics and Poisson regression modeling.

**Results:**

Of 6,741 publications identified, 239 met the inclusion criteria. Publications appeared in 93 journals and increased annually by 4%. Most studies were observational (72%) and conducted at academic centers (78%). Most protocols addressed diagnostic (45%) or diagnostic-therapeutic (28%) pathways, although there was a recent increasing trend in therapeutic pathways. Cardiology, particularly chest pain management, represented the predominant clinical focus, followed by infectious disease. Protocols describing geriatric care and viral illness management showed significant annual increases of 16% and 19%, respectively. Frequently reported outcomes included disposition rates, mortality, and return emergency department visits, whereas patient- and provider-centered outcomes were less commonly assessed.

**Conclusions:**

The EDOU protocol literature has expanded substantially over the past 25 years but remains concentrated in a limited number of clinical domains. Greater emphasis on underrepresented conditions and outcome measures may help guide the next generation of evidence-based EDOU protocols.

## 1 Introduction

### 1.1 Background

Amid rising national hospital occupancy and persistent staffing shortages, emergency department (ED) visits continue to increase, from 90 million in 1993 to 137 million in 2015, to 155 million in 2022.^1^, ^2^, ^3^, ^4^, ^5^, ^6^, ^7^, ^8^, ^9^ The challenge is compounded by restricted postacute care access, increasing admission rates among older adults with complex conditions, and system changes such as hospital closures and consolidation that reduce available inpatient capacity.^10^, ^11^, ^12^, ^13^, ^14^ Consequently, EDs frequently experience inpatient boarding as admitted patients wait for inpatient beds to become available. Boarding has been shown to contribute to ED overcrowding, prolonged wait times, increased medical errors, inflated treatment costs, and declining patient and physician satisfaction, ultimately leading to worse outcomes.^15^, ^16^, ^17^, ^18^, ^19^, ^20^

Emergency Department Observation Units (EDOUs) have emerged as a strategy to provide diagnostic and treatment services on a shorter timeline than a typical inpatient admission. EDOUs provide an opportunity to deliver time-limited, protocol-driven care for patients who are too high-risk for immediate discharge yet do not clearly require inpatient admission at the end of their ED visit. Typically located within or adjacent to the ED, EDOUs employ condition-specific protocols targeting patients with an expected length of stay of up to 2 midnights, after which patients are discharged or admitted as inpatients. Evidence shows that EDOUs improve operational efficiency, reduce overcrowding, lower costs, and enhance patient satisfaction.^21^, ^22^, ^23^, ^24^, ^25^

### 1.1 Importance

Initially introduced in the 1970s primarily for the evaluation of chest pain, EDOUs have evolved substantially since changes to Medicare observation billing policies in the early 2000s broadened their clinical applications.^26^ Today, nearly one-third of hospitals in the United States maintain operational EDOUs, supporting over 7 million observation visits annually as condition-specific protocols expand in volume and diversity.^27^^,^^28^ Despite this expansion, literature focusing specifically on EDOU protocols remains fragmented.

### 1.2 Goals of This Investigation

This scoping review aims to map the landscape of existing EDOU protocols and identify areas for further research. Specifically, we sought to (1) describe the types of EDOU protocols published to date, (2) characterize their associated outcomes, and (3) identify gaps in the evidence base to guide future research and clinical implementation.

## 2 Methods

### 2.1 Study Design

We followed the PRISMA-ScR (Preferred Reporting Items for Systematic Reviews and Meta-Analyses Extension for Scoping Reviews) reporting guidelines throughout the design and execution of our review.^29^ Our objective was to identify and characterize literature describing or evaluating condition-specific protocols implemented in EDOUs. A detailed description of the methodology is included in Supplementary Appendix 1. The search strategy was developed in collaboration with a medical reference librarian and the study authors.

### 2.2 Search Strategy

We searched the PubMed database for articles published between January 1, 2000, and June 1, 2025. Search terms captured 3 main concepts: (1) emergency medicine, (2) observation or clinical decision units, and (3) protocols, pathways, or algorithms. A detailed description of the search terms and strategy is available in Supplementary Appendix 2.

### 2.3 Selection of Articles

We included articles that described, implemented, or evaluated structured, condition-specific clinical protocols for adult populations (≥18 years) managed in EDOUs or analogous units, regardless of the various names used. For the purposes of this review, a “protocol” was defined as a clearly described, reproducible care pathway that specified a sequence of clinical actions, decision points, or interventions intended to guide patient management within the observation unit. Protocols could include diagnostic pathways, treatment algorithms, disposition plans, or multidisciplinary bundles. Excluded articles (1) described general observation practices, clinical trends, or unstructured management approaches without a defined pathway or algorithm, (2) focused exclusively on inpatient or non-ED settings, (3) only included pediatric patients, (4) were not written in English and could not be feasibly translated, (5) were primarily a nonsystematic review of established literature for educational purposes, or (6) did not have the full text of the manuscript available.

### 2.4 Screening & Data Extraction

We imported the titles and abstracts of the identified citations into Covidence systematic review software (Veritas Health Innovation, Melbourne, Australia) to facilitate the application of inclusion/exclusion criteria. Duplicate records were removed both automatically and manually. Two trained reviewers (MP, HC) independently conducted a preliminary screening of the titles and abstracts using the predefined inclusion and exclusion criteria. A senior reviewer (KS or AL) resolved disagreements through consensus. Full texts were then retrieved for all potentially eligible articles and uploaded into Covidence. At this stage, we excluded articles if the full text could not be located or if they were not written in English and could not be feasibly translated. All remaining full-text articles underwent a second round of independent screening by MP and HC to determine final eligibility. KS or AL resolved disagreements through consensus. A complete list of the included articles is available in Supplementary Appendix 3. Once the decision to include the article was finalized, MP and HC independently extracted data using the Covidence extraction tool described in Supplementary Appendix 4. Disagreements during the extraction phase were resolved by senior reviewer consensus. Inter-rater agreement for title/abstract and full-text screening was assessed, and detailed results are provided in Supplementary Appendix 5. Key data elements captured during extraction were: article type, publication journal and year, country in which a study was conducted, ED structure, type of protocol (diagnostic-only, combined diagnostic-therapeutic, or therapeutic-only), specialty of protocol (eg, cardiology, infectious disease), clinical conditions addressed (eg, chest pain, congestive heart failure, overdose), and any outcome metrics that were used to evaluate the protocol. The outcome metric categorizations used in this study were informed by a review of guidance published by the Agency for Healthcare Research and Quality (AHRQ).^30^

### 2.5 Data Analysis

Descriptive statistics were conducted at the level of individual publications and used to summarize the number of published EDOU protocols by year, geographic region, protocol type, associated specialty, clinical condition, and outcome measures evaluated. Counts and distributions were generated using Microsoft Excel and reflect the number of publications in which a protocol was described, rather than the number of unique protocols or institutions. To assess temporal trends in protocol publication, we used R to fit a series of unadjusted Poisson regression models with annual protocol count as the dependent variable and calendar year as the independent variable. We tested for overdispersion and planned to use negative binomial regression models as needed. Models were fit on protocols overall and stratified by protocol type and clinical condition. Results are reported as rate ratios with corresponding 95% confidence intervals, with statistical significance defined as a two-sided *P* value <.05.

## 3 Results

**Figure 1:**
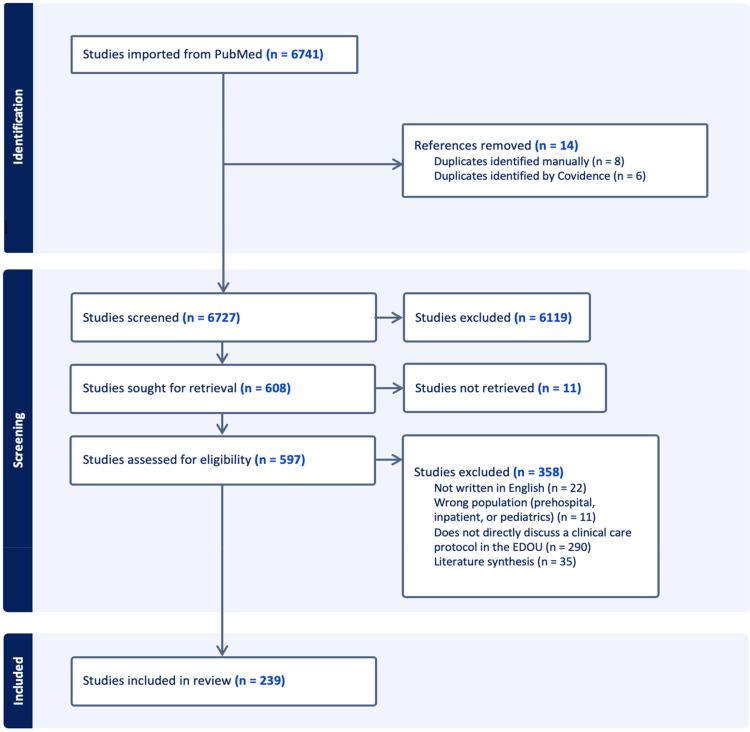
PRISMA flow diagram of articles reviewed for this study.

The search yielded 6741 records, of which 14 duplicates were removed, leaving 6727 unique records for screening. After title and abstract review, 608 full-text articles were assessed for eligibility, and 239 met the inclusion criteria and were included in the final analysis (Fig. 1). The most common reason for exclusion (79%; 290/369) was the absence of a clearly described reproducible care pathway or algorithm in the EDOU.

**Figure 2:**
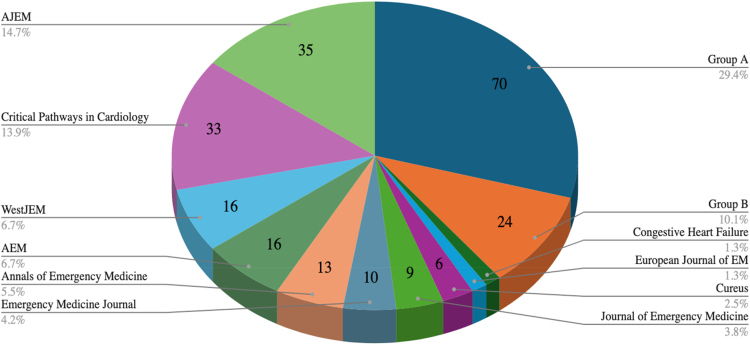
Distribution of article journals. This pie chart reflects the number of articles published in each journal during the study period. Group A refers to journals that published only one observation article included in this study. Group B comprises 12 journals, each published twice, for a total of 24 articles. The complete list of included journals is provided in Appendix 6. AJEM, American Journal of Emergency Medicine; WestJEM, Western Journal of Emergency Medicine; AEM, Academic Emergency Medicine.

Publications appeared in 93 journals, though most (83/93) contributed fewer than 3 articles (Fig. 2). The *American Journal of Emergency Medicine* (n = 35) and *Critical Pathways in Cardiology* (n = 33) were the most prolific, accounting for 28% (68/239) of all included articles. By design, most articles were observational studies (n = 173), followed by nonrandomized experimental studies (n = 25), randomized controlled trials (n = 12), economic evaluations (n = 7), commentaries or opinion pieces (n = 6), qualitative studies (n = 5), systematic or scoping reviews (n = 4), and case reports or series (n = 3) (Table 1). Geographically, most articles originated from the United States (n = 188), followed by Europe (excluding the United Kingdom, n = 16) and Asia (n = 16). The majority of research (78%; 187/239) was conducted in academic hospitals, with the remainder conducted in urban, community, or mixed settings. Patient populations were predominantly general adults (93%, 222/239), with only 3% (7/239) specifying nongeriatric adults, and 4% (10/239) focusing on geriatric populations.

**Table 1:** Frequency of study designs for published articles.

Study Design	Count
Observational study (cohort, cross-sectional, case-control)	173
Nonrandomized experimental study	25
Randomized controlled trial	12
Systematic or scoping review	4
Qualitative research	5
Case report or series	3
Economic evaluation	7
Commentary or opinion	6
Other	4

**Figure 3:**
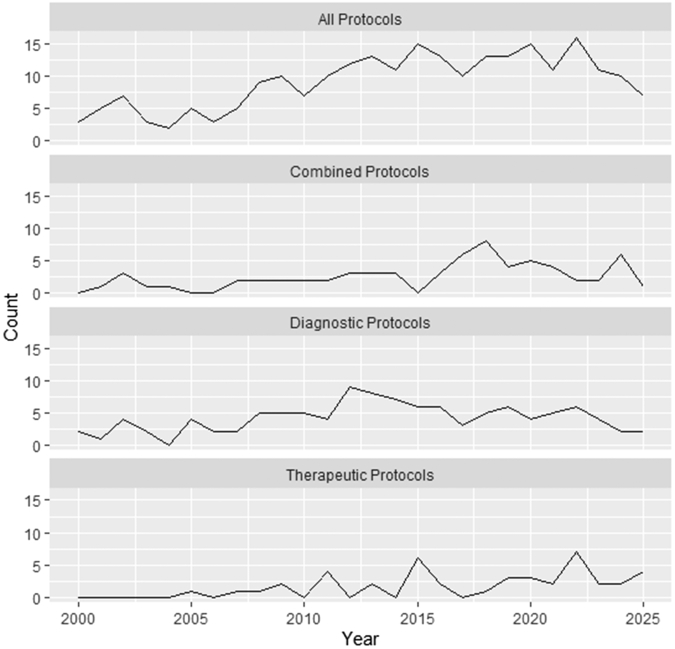
Articles and protocol types published per year. This figure displays the annual number of published articles describing EDOU protocols over the study period, stratified by protocol type. EDOU, Emergency Department Observation Unit.

Poisson regression models were implemented, and there was no evidence of significant overdispersion. Included articles were published between 2000 and 2025, averaging 9 publications per year with an approximate increase of 4% per year [rate ratio (RR) 1.04, 95% CI 1.03 to 1.06, *P*<.001]. Among the 239 included articles, most described diagnostic-only protocols (45%; 109/239), whereas 28% (66/239) described combined diagnostic-therapeutic protocols, and 18% (43/239) described therapeutic-only protocols. Protocols characterized as therapeutic or combined diagnostic-therapeutic were found to have statistically significant annual increases of approximately 10% (RR 1.10, 95% CI 1.05 to 1.16, *P*<.001) and 6% (RR 1.06, 95% CI 1.02 to 1.10, *P*<.001), respectively (Fig. 3).

**Figure 4:**
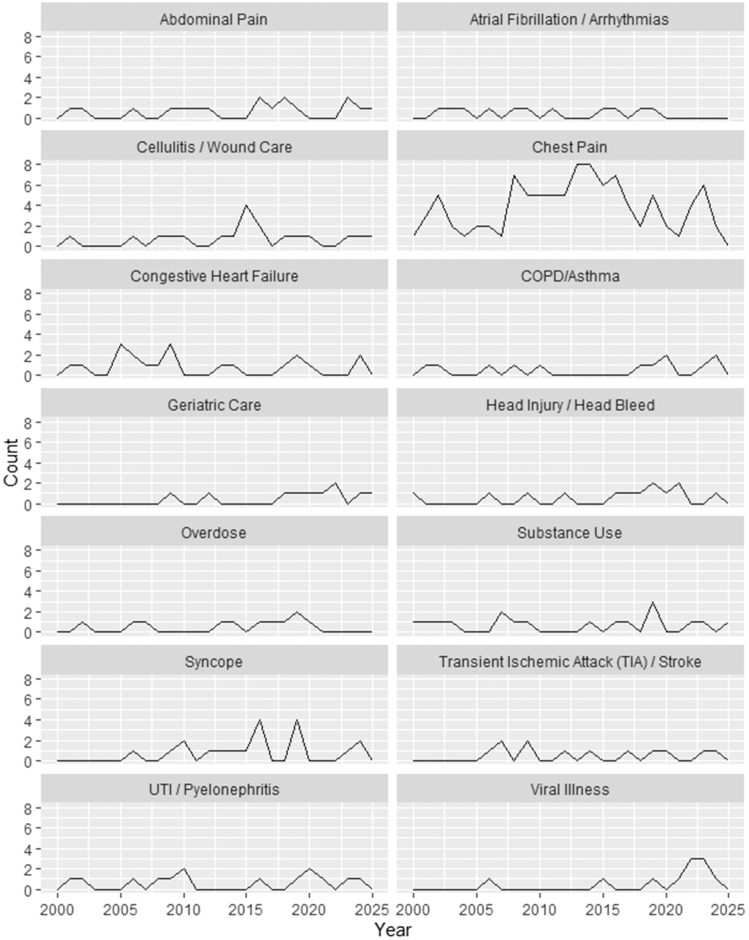
Selected condition-specific protocols over time. This figure presents the number of articles published per year describing EDOU protocols for clinical conditions with at least 10 mentions across the literature over the study period.

**Figure 5:**
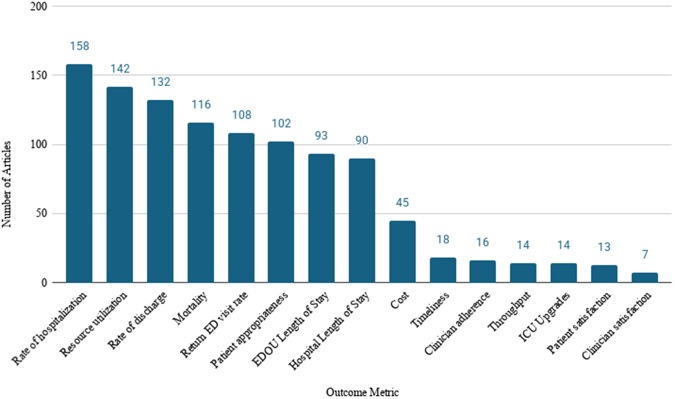
Distribution of outcome metrics in published EDOU protocol literature. This figure illustrates the distribution of outcome metrics reported in studies of EDOU protocols. Each bar represents the number of studies assessing a given outcome. Individual articles may contribute to multiple outcome categories, and, therefore, counts are not mutually exclusive. PE, pulmonary embolism; EDOU, Emergency Department Observation Unit; ICU, intensive care unit.

Protocols addressed a wide range of clinical conditions and specialties, with cardiology being most frequently represented (134 articles), followed by infectious disease (34 articles). Chest pain was the most common indication (99 articles). The next most common indications were congestive heart failure (20 articles), followed by cellulitis/wound care (19), syncope (19), abdominal pain (17), substance use (17), and UTI / pyelonephritis (14 articles). Table 2 presents the complete list of protocol conditions and their respective frequencies in the literature. Among the conditions that appeared at least 10 times, geriatric care and viral illness protocols were found to significantly increase over the study period by approximately 16% per year (RR 1.16, 95% CI 1.03 to 1.29, *P*<.05) and 19% per year [RR 1.19, 95% CI 1.06 to 1.34, *P*<.01], respectively (Fig. 4). Outcomes evaluated varied considerably across articles (Fig. 5). The most frequently assessed outcomes were hospitalization rate (158), discharge rate (132), mortality (116), and return ED visit rate (108).

**Table 2:** Frequency of clinical condition protocols across the literature. Each count represents the number of publications describing a protocol for the specified clinical condition. Articles could contribute to multiple condition categories if more than one protocol or indication was reported. Conditions displayed on the same line are presented for formatting purposes only and do not represent grouped categories.

Clinical Condition	Number of Articles
Chest pain	99
Congestive heart failure	20
Cellulitis/Wound care Syncope (including near-syncope)	19
Abdominal pain Substance Use (including alcohol)	17
UTI/Pyelonephritis	14
Head injury/Head bleed	13
COPD/Asthma Transient ischemic attack (TIA)/Stroke	12
Atrial fibrillation (or other arrhythmias) Overdose Viral illness (including COVID)	11
Geriatric care	10
Back pain Gastroenteritis	9
Psychiatric conditions Dehydration / Nausea & vomiting Social interventions / Case management	8
VTE (DVT & PE) Virtual care / Telehealth Musculoskeletal complaints (excluding back pain)	7
Telemetry/Vital sign monitoring Pneumonia Dysglycemia Cancer management	6
Physical therapy/Rehab placement Allergic reaction	5
Vertigo / Dizziness Sickle cell pain Headache / Migraine GI bleed (upper and lower)	4
SBO/Ileus Pancreatitis Anemia Transfusion Seizure Renal colic/Kidney stones Electrolyte abnormalities Dental / ENT management	3
Pneumothorax	2
Palliative care / Hospice CO exposure Burn	1

UTI, urinary tract infection; TIA, transient ischemic attack; COPD, chronic obstructive pulmonary disease; COVID, infectious respiratory disease caused by the SARS-CoV-2 virus; VTE, venous thromboembolism; DVT, deep vein thrombosis; PE, pulmonary embolism; GI, gastrointestinal; SBO, small bowel obstruction; ENT, ear, nose & throat; CO, carbon monoxide.

## 4 Limitations

This review has several limitations that should be considered when interpreting these findings. The search was restricted to PubMed-indexed articles and to studies published in or translated into English, which may have excluded relevant literature from other databases or non-English sources. However, a supplementary Embase search identified minimal additional eligible studies, suggesting limited impact on overall findings. The search window (January 1, 2000 to June 1, 2025) may have omitted both earlier protocols that are no longer under study and more recent protocols that have not yet been published. Because our aim was to provide a descriptive mapping of the literature, we did not assess study quality or risk of bias. In addition, our analysis was restricted to published protocols, while many observation units likely rely on internal, institution-specific pathways that are not publicly reported, introducing potential publication bias.

Furthermore, our inclusion criteria focused on explicitly defined, condition-specific observation unit protocols. As a result, this review does not capture a substantial body of literature on risk stratification tools that inform observation unit care but are not presented as formalized protocols. This is particularly relevant in areas such as chest pain evaluation, where risk stratification frameworks play a central role in guiding observation unit management but may be embedded within clinical workflows rather than described as discrete protocols.

There are also limitations related to how protocols and outcomes were represented in the available literature. Multiple publications may describe the same protocol from a single institution, which limits the ability to estimate the number of unique protocols. Conversely, distinct protocols for the same condition across institutions could not always be differentiated using available data. As a result, counts of clinical conditions and protocol types reflect the frequency of publications rather than unique protocols, which may overrepresent commonly studied conditions such as chest pain. In addition, because individual studies frequently reported multiple protocols and outcomes, one-to-one associations between specific protocols and outcome measures could not be established.

Finally, the outcome measures reported across studies were heavily weighted toward operational metrics, such as disposition and resource utilization. This limits insight into the effects of observation unit protocols on patient-centered outcomes, including patient experience, functional status, and quality of life, which remain underrepresented in the current literature.

## 5 Discussion

This scoping review provides a comprehensive overview of published literature on EDOU protocols over the past 25 years. We identified 239 articles describing or evaluating condition-specific protocols, most of which emphasized diagnostic or combined diagnostic-therapeutic approaches. Across the articles, hospital admission and discharge disposition emerged as dominant operational outcome metrics, reflecting the central role of EDOUs in optimizing patient flow and minimizing low-yield inpatient admissions. Chest pain protocols were by far the most prevalent, consistent with the historical foundation of EDOUs and their ongoing importance in cardiac evaluation.^31^^,^^32^

High-frequency cardiac conditions comprised a large share of reported protocols, most commonly chest pain, syncope, congestive heart failure, and arrhythmias. Their prominence reflects high ED prevalence, elevated medicolegal risk, diagnostic uncertainty, and the time-sensitive nature of associated pathology.^33^, ^34^, ^35^, ^36^, ^37^, ^38^ These conditions are well suited to protocolized care because they often require serial examinations, cardiac biomarkers, continuous monitoring, or advanced imaging, all of which can be delivered during short EDOU or brief inpatient stays. This risk profile has supported the development of specialized chest pain units as distinct operational models within observation units, designed to accelerate evaluation while improving safety and reducing unnecessary admissions. In 2006, chest pain was the second most common ED complaint, with nearly 19% of visits resulting in hospital admission, compared with 12.8% of all ED presentations.^39^ By 2016, admission rates for chest pain declined to 3.9%. Collectively, the volume and consistency of cardiac-focused protocols underscore how observation units have evolved to manage high-risk, high-volume presentations.

In contrast, several common ED presentations were infrequently represented in the observation unit literature despite substantial clinical burden. Palliative care appeared in only 1 study, whereas seizures and kidney stones were each described in 3 studies, and headache and dizziness, two of the most common ED complaints, appeared in only 4 articles each. This contrasts with national visit volumes of ∼1.6 million for seizure, 1.2 million for kidney stones, 2.6 million for dizziness, and 6 million for headache annually.^40^, ^41^, ^42^, ^43^ Many of these conditions require serial assessments, diagnostic imaging, or time-based therapies that align well with observation unit capabilities. The reasons for this mismatch are unclear. Some conditions may be managed within broader observation pathways without being explicitly named, while variation in triage practices, limited evidence-based risk stratification, and local care models may influence whether patients are observed, discharged, or admitted. Lower-risk presentations may be discharged directly, whereas higher-risk cases, particularly seizures requiring rapid intervention or airway management, may be routed to higher-acuity settings that observation units seek to avoid. In contrast to chest pain, where validated tools such as the HEART score support protocol development, comparable risk stratification frameworks are limited for many of these conditions. Together, these findings identify gaps in the observation unit literature relative to clinical volume and highlight the need for further study to clarify whether these gaps reflect underdevelopment, underreporting, or differences in observation unit utilization.

Temporal trends further illustrate the evolution of observation medicine. Publications related to EDOUs increased steadily over the 25-year study period, reflecting growing clinical adoption and operational integration. Chest pain remained the dominant indication throughout, consistent with its foundational role in observation care, even after the introduction of high-sensitivity troponin assays in 2017.^44^ In contrast, geriatric care and viral illness protocols showed significant growth over time, with viral illness protocols rising sharply from 2021 to 2025, likely in response to the COVID-19 pandemic. Together, these trends highlight both the durability of core observation unit indications and the capacity of observation units to adapt rapidly to emerging clinical and public health demands.

Diagnostic protocols have consistently outnumbered therapeutic protocols, although both increased over time. This predominance reflects the traditional role of observation units in evaluating diagnostically uncertain conditions such as chest pain and syncope. More recently, rising healthcare costs, policy pressures, and limited outpatient access have expanded the diagnostic role of observation units for patients who might otherwise undergo outpatient evaluation. In particular, increasing reliance on managed care models and Medicare Advantage plans has introduced additional barriers to timely outpatient diagnostics, including prior authorization requirements and restricted network access, which can delay care and shift time-sensitive evaluations back into the acute care setting. Concurrently, worsening ED boarding and inpatient crowding have driven hospitals to expand therapeutic capabilities within observation units. From an operational standpoint, expanding observation unit services often leverages existing hospital-based infrastructure, such as access to stress testing, advanced imaging, and specialty consultation, rather than requiring the development of new outpatient programs, which can be resource-intensive and slower to implement. Interventions such as diuresis, cardioversion, and anticoagulation, once reserved for inpatient settings, are now commonly initiated and monitored in these units.^45^, ^46^, ^47^, ^48^ This shift was further accelerated during the COVID-19 pandemic, when diagnosis became relatively streamlined while the need for treatment and short-term monitoring increased.^49^ Together, these trends reflect the evolution of observation units from a primarily diagnostic setting to a flexible, multidisciplinary space that bridges emergency and inpatient care.

Across included articles, outcome measures focused predominantly on operational performance. Discharge rates, hospitalization rates, and resource utilization were most frequently reported, reflecting the emphasis on throughput and efficiency as central goals of observation medicine. While these metrics align with efforts to reduce ED crowding and preserve inpatient capacity, outcomes such as clinician satisfaction, patient-reported experience, and escalation of care were assessed far less often. This imbalance limits understanding of how observation unit protocols affect clinician experience, patient safety, and patient-centered outcomes beyond operational efficiency.

Some studies describe the breadth of protocols used for specific conditions without evaluating their comparative effectiveness. To date, the observation unit literature has focused more on demonstrating overall efficacy than on determining which protocols work best for specific patient populations. Comparative evaluation would be facilitated by standardized, multidimensional outcome measures that better capture both clinical and operational impact. Such an approach could accelerate protocol refinement for well-studied cardiac conditions and support more rigorous development of protocols for underrepresented conditions, such as headache and seizure.

In conclusion, this scoping review demonstrates the expansion and diversification of EDOU protocols over the past 25 years. Diagnostic pathways, particularly for chest pain, remain predominant, but interest in structured observation care for a wider range of conditions is increasing. However, the existing literature remains heavily focused on operational efficiency metrics, with comparatively limited attention to patient-centered outcomes. Future research should target underrepresented conditions, adopt standardized outcome measures, highlight patient-centered and clinician-centered outcomes (including patient experience, safety, and quality of life), and incorporate multicenter designs to inform best practices and support evidence-based development of EDOU protocols.

## Author Contributions

KDS, ADL, MP, HC, and CWB developed the manuscript concept and implemented the study design. MAM performed quantitative data analysis. KDS, ADL, MP, HC, JJB, CTB, MAM, DAM, and CWB all provided critical revisions of the manuscript.

## Funding and Support

This work was supported by the Brigham-TechFoundation Remote Data Science & Medical Research Internship.

## Declaration of Generative AI and AI-Assisted Technologies in the Writing Process

During the preparation of this work the authors used ChatGPT by OpenAI in order to refine the text of the manuscript and improve clarity. After using this tool/service, the authors reviewed and edited the content as needed and take full responsibility for the content of the published article.

## Conflict of Interest

Dr. Christopher Baugh is an editor for *JACEP Open*. Otherwise, all authors report no further conflicts of interest.
