# Supplementary material for: Condition-Specific Protocols Used in the Emergency Department Observation Unit: A Scoping Review
Source: J Am Coll Emerg Physicians Open. 2026 May 27;7(4):100425. doi: 10.1016/j.acepjo.2026.100425 (PMC13234234; doi:10.1016/j.acepjo.2026.100425)
Supplement: Supplementary Appendix [file mmc1.docx]

**Appendix**

Appendix 1: Emergency Department Observation Units: A Scoping Review Protocol & Methodology

*Review Questions:* What research exists in the field of observation medicine regarding condition-specific protocols? What is the study design of such research, and in what context of the hospital system does such research occur? There are two sub-questions to these general questions. The first asks what field of medicine and which condition the observation medicine protocol focuses on. The second asks which outcomes are measured and recorded for such protocols, and why they are recorded.

*Key Words:* Observation Unit; Emergency Department; Clinical Protocols; Accelerated Diagnostic Pathways; Scoping Review; Clinical Observation Units

*Objective:* The purpose of this scoping review is to identify research on Emergency Department Observation Units (EDOUs) that describes clinical protocols, patient outcomes, and unit functions within the broader hospital system. The goal is to map the current literature, identify the types of clinical protocols described, and highlight the research gaps.

*Rationale:* Published research on condition-specific EDOU protocols is limited. The existing literature is scattered across specialties such as cardiology, neurology, and emergency medicine, with no comprehensive evaluation of these protocols as a whole. There is no centralized overview of the types of EDOU protocols, the conditions they target, or the outcomes measured. This scoping review aims to address that gap by synthesizing and analyzing existing research on condition-specific protocols in observation medicine, identifying evidence gaps, and guiding future research in the field. Specifically, this review seeks to describe the types of protocols published in the literature, the extent to which they have been studied, and the methods used to examine them. A scoping review is the most appropriate approach for this topic. The literature on EDOUs is broad and diverse, spanning multiple specialties. Rather than answering a single, narrow question, this review will map what is known and identify knowledge gaps. The findings will provide a foundation for future studies and help clinicians and researchers better understand how EDOUs function within the broader hospital system.

*Concept:*This review focuses on adult patients (≥18 years) managed under observation status within EDOUs or closely analogous observation units. To be included, studies were required to describe condition-specific protocols guiding patient care within these units, rather than general or unstructured observation practices. While observation medicine may be practiced in a variety of clinical settings, this review is specifically focused on observation units that are operationally and structurally aligned with EDOUs, including units located within or adjacent to the emergency department and designed to provide time-limited, protocol-driven care*.*

*Context:*Observation medicine refers to the short-term evaluation and management of patients who do not meet criteria for acute hospitalization or inpatient admission but require additional monitoring, diagnostic testing, or treatment prior to disposition. This review specifically examines condition-specific protocols implemented within EDOUs or similar unit-based observation settings. Only articles with a full text available and published in English or translated into English are included. This was done for practical reasons, and given the dominance of the English language in academia, it is expected to yield an accurate representation of the entire body of literature.

*Types of Sources:*The purpose of this review was to capture an extensive breadth of research and publications in the field of observation medicine, considering all the new applications and protocols that have been developed to enhance treatment in a variety of fields. This review considers study designs, including observational studies, non-randomized experimental studies, randomized controlled trials, systematic or scoping reviews, qualitative research, case reports, economic evaluations, and commentaries.

*Study Design:* A comprehensive PubMed search will be conducted from January 1, 2000, to June 1st, 2025. Eligible studies will be screened for relevance to EDOU clinical protocols. Data will be extracted on institution, study design, patient populations, clinical conditions, specialties involved, and outcomes measured. Findings will be synthesized to describe the current state of research and highlight areas requiring further study.

*Inclusion & Exclusion Criteria:* This review includes all studies describing Emergency Department Observation Units that focus on clinical protocols, structure, function, or outcomes. Studies must involve adult or mixed patient populations admitted under observation status. Articles comparing multiple EDOUs or describing specific clinical pathways (for example, chest pain, asthma, or TIA) will also be included. Pediatric and obstetric populations will be excluded. Only studies with available full texts that are published in English or already translated into English from January 1, 2000, to June 1st, 2025, will be considered.

*Study/Source of Evidence Selection:* After the search, all the identified citations were collated and uploaded into Covidence. Two independent reviewers then screened the titles and abstracts to assess whether the paper of interest discussed condition-specific protocols implemented within EDOUs or similar unit-based observation settings in adult (≥18 years) patient populations. Full texts were then found and uploaded to Covidence. The full-text review included screening articles for discussion of condition-specific protocols within observation medicine. Reasons for excluding full-text sources were recorded and reported in the scoping review. Any disagreements that arose between reviewers at each stage of the selection process were resolved through discussion and under the guidance of additional reviewers.

*Data Extraction:* Data will be extracted from all papers included in the scoping review by at least two independent reviewers using a customized data extraction tool developed in Covidence. The extracted information will consist of details on participants, the concept, the context, the study methods, and key findings relevant to the review questions. A draft extraction form is provided in Appendix 4. This form was initially developed in Covidence and revised as necessary throughout the extraction process. Any modifications were applied consistently across all included papers.

Appendix 2: Search Strategy

| **Concept** | **Search Strategy** |
| --- | --- |
| Emergency Medicine | (Emergency[tiab] OR “Emergency Service, Hospital”[MESH] OR “Evidence-Based Emergency Medicine”[MESH] OR “Emergency Medicine”[MESH] OR “Emergency Department”[MESH]) |
| Observation Unit Protocols | (“Observation Units”[MESH] OR “Observation unit”[tiab] OR “Observation units”[tiab] OR “observation medicine”[tiab] OR “emergency department observation”[tiab] OR “EDOU”[tiab] OR “clinical protocol”[tiab] OR “clinical protocols”[tiab] OR “care pathway”[tiab] OR “care pathways”[tiab] OR “clinical pathway”[tiab] OR “clinical pathways”[tiab] OR “standardized protocol”[tiab] OR “structured protocol”[tiab] OR “observation protocol”[tiab] OR “observation protocols”[tiab]) OR “accelerated diagnostic protocol”[tiab] OR “accelerated diagnostic protocol”[tiab] OR “outpatient pathway”[tiab] or “outpatient protocol”[tiab] OR “treatment pathway”[tiab] OR “treatment pathways”[tiab] OR “CDU”[tiab] OR “Clinical Decision Unit”[tiab]) |
| Time Frame | (2000:2025[pdat]) |

Appendix 3: Included References

Abbass I. “Variability in the Initial Costs of Care and One-Year Outcomes of Observation Services.” *West J Emerg Med* (United States) 16, no. 3 (2015): 395–400. <https://doi.org/10.5811/westjem.2015.2.24281>.

Abbass IM, Krause TM, Virani SS, Swint JM, Chan W, and Franzini L. “Revisiting the Economic Efficiencies of Observation Units.” *Manag Care* (United States) 24, no. 3 (2015): 46–52.

Abbass IM, Virani SS, Michael Swint J, Chan W, and Franzini L. “One-Year Outcomes Associated with Using Observation Services in Triaging Patients with Nonspecific Chest Pain.” *Clin Cardiol* (United States) 37, no. 10 (2014): 591–96. <https://doi.org/10.1002/clc.22319>.

Abetz JW, Adams NG, and Mitra B. “Skin and Soft Tissue Infection Management Failure in the Emergency Department Observation Unit: A Systematic Review.” *Emerg Med J* (England) 35, no. 1 (2018): 56–61. <https://doi.org/10.1136/emermed-2016-205950>.

Abiri A and Tucker P. “Advancing Telemedicine: Virtual Rounding and Care in an Emergency Department Observation Unit.” *Adv Emerg Nurs J* (United States) 47, no. 1 (2025): 80–84. <https://doi.org/10.1097/TME.0000000000000553>.

Afacan MA and Tayfur İ. “Comparison of the Effects of Metoclopramide and Ondansetron on Emergency Service Observation Times in Acute Gastroenteritis-Related Nausea and Vomiting Cases.” *Sisli Etfal Hastan Tip Bul* (Turkey) 53, no. 2 (2019): 186–89. <https://doi.org/10.14744/SEMB.2019.80217>.

Amirian J, Javdan O, Misher J, et al. “Comparative Efficiency of Exercise Stress Testing with and without Stress-Only Myocardial Perfusion Imaging in Patients with Low-Risk Chest Pain.” *J Nucl Cardiol* (United States) 25, no. 4 (2018): 1274–82. <https://doi.org/10.1007/s12350-016-0774-y>.

Anderson K, Goldsmith LP, Lomani J, et al. “Short-Stay Crisis Units for Mental Health Patients on Crisis Care Pathways: Systematic Review and Meta-Analysis.” *BJPsych Open* (England) 8, no. 4 (2022): e144. <https://doi.org/10.1192/bjo.2022.534>.

Anderson KL, Limkakeng A, Damuth E, and Chandra A. “Cardiac Evaluation for Structural Abnormalities May Not Be Required in Patients Presenting with Syncope and a Normal ECG Result in an Observation Unit Setting.” *Ann Emerg Med* (United States) 60, no. 4 (2012): 478-84.e1. <https://doi.org/10.1016/j.annemergmed.2012.04.023>.

Antwi-Amoabeng D, Roongsritong C, Taha M, et al. “SVEAT Score Outperforms HEART Score in Patients Admitted to a Chest Pain Observation Unit.” *World J Cardiol* (United States) 14, no. 8 (2022): 454–61. <https://doi.org/10.4330/wjc.v14.i8.454>.

Aplin KS, Coutinho McAllister S, Kupersmith E, and Rachoin JS. “Caring for Patients in a Hospitalist-Run Clinical Decision Unit Is Associated with Decreased Length of Stay without Increasing Revisit Rates.” *J Hosp Med* (United States) 9, no. 6 (2014): 391–95. <https://doi.org/10.1002/jhm.2188>.

Arthur CL and Greenawald MH. “12-Hour Protocol Safe for Cocaine-Associated Chest Pain.” *J Fam Pract* (United States) 52, no. 6 (2003): 452–54.

Ashburn NP, Snavely AC, Paradee BE, O’Neill JC, Stopyra JP, and Mahler SA. “Age Differences in the Safety and Effectiveness of the HEART Pathway Accelerated Diagnostic Protocol for Acute Chest Pain.” *J Am Geriatr Soc* (United States) 70, no. 8 (2022): 2246–57. ClinicalTrials.gov/NCT02056964. <https://doi.org/10.1111/jgs.17777>.

Ashburn NP, Snavely AC, Rikhi R, et al. “Rarely Tested or Treated but Highly Prevalent: Hypercholesterolemia in ED Observation Unit Patients with Chest Pain.” *Am J Emerg Med* (United States) 71 (2023): 47–53. <https://doi.org/10.1016/j.ajem.2023.06.009>.

Ashburn NP, Snavely AC, Rikhi RR, et al. “Chest Pain Observation Unit: A Missed Opportunity to Initiate Smoking Cessation Therapy.” *Am J Emerg Med* (United States) 68 (2023): 17–21. <https://doi.org/10.1016/j.ajem.2023.02.033>.

Ashburn NP, Snavely AC, Stanek LS, et al. “Emergency Department Observation Unit Patients Want Evaluation and Treatment for Hypercholesterolemia: A Health Belief Model Study.” *Crit Pathw Cardiol* (United States) 22, no. 3 (2023): 91–94. <https://doi.org/10.1097/HPC.0000000000000324>.

Asher E, Reuveni H, Shlomo N, et al. “Clinical Outcomes and Cost Effectiveness of Accelerated Diagnostic Protocol in a Chest Pain Center Compared with Routine Care of Patients with Chest Pain.” *PLoS One* (United States) 10, no. 1 (2015): e0117287. <https://doi.org/10.1371/journal.pone.0117287>.

Aslam MI, Martin-Ucar AE, Nakas A, and Waller DA. “Surgical Management of Pneumothorax: Significance of Effective Admission or Communication Strategies between the District General Hospitals and Specialized Unit.” *Interact Cardiovasc Thorac Surg* (England) 13, no. 5 (2011): 494–98. <https://doi.org/10.1510/icvts.2011.277707>.

Balai E, Bhamra N, Gupta K, Jolly K, and Barraclough J. “Implementation of an Acute Tonsillitis Management Protocol within a Clinical Decisions Unit.” *Ann R Coll Surg Engl* (England) 103, no. 9 (2021): 690–93. <https://doi.org/10.1308/rcsann.2021.0060>.

Baugh CW, Blankstein R, Ganguli I, et al. “Frequency, Compliance, and Yield of Cardiac Testing after High-Sensitivity Troponin Accelerated Diagnostic Protocol Implementation.” *Am J Emerg Med* (United States) 72 (2023): 64–71. <https://doi.org/10.1016/j.ajem.2023.07.014>.

Baugh CW, Cash RE, Meguerdichian D, et al. “An Electronic Medical Record Intervention to Increase Pharmacologic Prophylaxis for Venous Thromboembolism in Emergency Department Observation Patients.” *Ann Emerg Med* (United States) 83, no. 1 (2024): 24–34. <https://doi.org/10.1016/j.annemergmed.2023.08.017>.

Baugh CW, Clark CL, Wilson JW, et al. “Creation and Implementation of an Outpatient Pathway for Atrial Fibrillation in the Emergency Department Setting: Results of an Expert Panel.” *Acad Emerg Med* (United States) 25, no. 9 (2018): 1065–75. <https://doi.org/10.1111/acem.13410>.

Baugh CW, Epstein LM, Schuur JD, et al. “Atrial Fibrillation Emergency Department Observation Protocol.” *Crit Pathw Cardiol* (United States) 14, no. 4 (2015): 121–33. <https://doi.org/10.1097/HPC.0000000000000057>.

Baugh CW, Liang LJ, Probst MA, and Sun BC. “National Cost Savings from Observation Unit Management of Syncope.” *Acad Emerg Med* (United States) 22, no. 8 (2015): 934–41. <https://doi.org/10.1111/acem.12720>.

Beauchamp GA, Hart KW, Lindsell CJ, et al. “Performance of a Multi-Disciplinary Emergency Department Observation Protocol for Acetaminophen Overdose.” *J Med Toxicol* (United States) 9, no. 3 (2013): 235–41. <https://doi.org/10.1007/s13181-013-0310-2>.

Beigel R, Fardman A, Goldkorn R, et al. “Feasibility and Safety of Evaluating Patients with Prior Coronary Artery Disease Using an Accelerated Diagnostic Algorithm in a Chest Pain Unit.” *PLoS One* (United States) 11, no. 9 (2016): e0163501. <https://doi.org/10.1371/journal.pone.0163501>.

Bellew SD, Bremer ML, Kopecky SL, et al. “Impact of an Emergency Department Observation Unit Management Algorithm for Atrial Fibrillation.” *J Am Heart Assoc* (England) 5, no. 2 (2016). <https://doi.org/10.1161/JAHA.115.002984>.

Berger D, King S, Caldwell C, et al. “Returns After Discharge From the Emergency Department Observation Unit: Who, What, When, and Why?” *West J Emerg Med* (United States) 24, no. 3 (2023): 390–95. <https://doi.org/10.5811/westjem.59023>.

Berlyand Y, Baugh JJ, Lee AH, et al. “Evaluation of a COVID-19 Emergency Department Observation Protocol.” *Am J Emerg Med* (United States) 56 (2022): 205–10. <https://doi.org/10.1016/j.ajem.2022.02.034>.

Bledsoe JR, Woller SC, Stevens SM, et al. “Cost-Effectiveness of Managing Low-Risk Pulmonary Embolism Patients without Hospitalization. The Low-Risk Pulmonary Embolism Prospective Management Study.” *Am J Emerg Med* (United States) 41 (2021): 80–83. <https://doi.org/10.1016/j.ajem.2020.12.042>.

Bock BC, Becker BM, Niaura RS, Partridge R, Fava JL, and Trask P. “Smoking Cessation among Patients in an Emergency Chest Pain Observation Unit: Outcomes of the Chest Pain Smoking Study (CPSS).” *Nicotine Tob Res* (England) 10, no. 10 (2008): 1523–31. <https://doi.org/10.1080/14622200802326343>.

Borczuk P, Van Ornam J, Yun BJ, Penn J, and Pruitt P. “Rapid Discharge After Interfacility Transfer for Mild Traumatic Intracranial Hemorrhage: Frequency and Associated Factors.” *West J Emerg Med* (United States) 20, no. 2 (2019): 307–15. <https://doi.org/10.5811/westjem.2018.12.39337>.

Brown MD, Reeves MJ, Glynn T, Majid A, and Kothari RU. “Implementation of an Emergency Department Based Transient Ischemic Attack Clinical Pathway: A Pilot Study in Knowledge Translation.” *Acad Emerg Med* (United States) 14, no. 11 (2007): 1114–19. <https://doi.org/10.1197/j.aem.2007.04.019>.

Budde J, Agarwal P, Mazumdar M, Yeo J, and Braman SS. “Can an Emergency Department Observation Unit Reduce Hospital Admissions for COPD Exacerbation?” *Lung* (United States) 196, no. 3 (2018): 267–70. <https://doi.org/10.1007/s00408-018-0102-1>.

Burkhardt J, Peacock WF, and Emerman CL. “Predictors of Emergency Department Observation Unit Outcomes.” *Acad Emerg Med* (United States) 12, no. 9 (2005): 869–74. <https://doi.org/10.1197/j.aem.2005.03.534>.

Carlberg M, Lum M, Ojcius J, et al. “Prevalence and Significance of Renal Dysfunction Among Emergency Department Observation Patients With Chest Pain.” *Crit Pathw Cardiol* (United States) 18, no. 4 (2019): 185–88. <https://doi.org/10.1097/HPC.0000000000000178>.

Carpenter JE, Short N, Williams TE, Yandell B, and Bowers MT. “Improving Congestive Heart Failure Care with a Clinical Decision Unit.” *Nurs Econ* (United States) 33, no. 5 (2015): 255–62.

Caspers C, Smith SW, Seth R, Femia R, and Goldfrank LR. “Observation Services Linked With an Urgent Care Center in the Absence of an Emergency Department: An Innovative Mechanism to Initiate Efficient Health Care Delivery in the Aftermath of a Natural Disaster.” *Disaster Med Public Health Prep* (United States) 10, no. 3 (2016): 405–10. <https://doi.org/10.1017/dmp.2016.49>.

Cassin M, Macor F, Cappelletti P, et al. “Management of Patients with Low-Risk Chest Pain at the Time of Admission: A Prospective Study on a Non-Selected Population from the Emergency Department.” *Ital Heart J* (Italy) 3, no. 7 (2002): 399–405.

Chaftari P, Lipe DN, Wattana MK, et al. “Outcomes of Patients Placed in an Emergency Department Observation Unit of a Comprehensive Cancer Center.” *JCO Oncol Pract* (United States) 18, no. 4 (2022): e574–85. <https://doi.org/10.1200/OP.21.00478>.

Chandra A, Compton S, Sochor M, Puri S, and Zalenski RJ. “Untreated Hypercholesterolemia in an Emergency Department Chest Pain Observation Unit Population.” *Acad Emerg Med* (United States) 9, no. 7 (2002): 699–702. <https://doi.org/10.1111/j.1553-2712.2002.tb02148.x>.

Chang AM, Shofer FS, Weiner MG, et al. “Actual Financial Comparison of Four Strategies to Evaluate Patients with Potential Acute Coronary Syndromes.” *Acad Emerg Med* (United States) 15, no. 7 (2008): 649–55. <https://doi.org/10.1111/j.1553-2712.2008.00159.x>.

Chavez J, Srinivasan A, Ely S, et al. “Thrombolysis in Myocardial Infarction Risk Score in an Observation Unit Setting.” *Crit Pathw Cardiol* (United States) 12, no. 3 (2013): 137–40. <https://doi.org/10.1097/HPC.0b013e3182998bc1>.

Cheema MA, Abdullah HMA, Ullah W, et al. “Role of Echocardiography in Diagnostic Evaluation of Patients Admitted to Observation Unit.” *Am J Cardiovasc Dis* (United States) 9, no. 6 (2019): 127–33.

Cheng AHY, Barclay NG, and Abu-Laban RB. “Effect of a Multi-Diagnosis Observation Unit on Emergency Department Length of Stay and Inpatient Admission Rate at Two Canadian Hospitals.” *J Emerg Med* (United States) 51, no. 6 (2016): 739-747.e3. <https://doi.org/10.1016/j.jemermed.2015.12.024>.

Chong E, Zhu B, Ng SHX, et al. “Emergency Department Interventions for Frailty (EDIFY): Improving Functional Outcomes in Older Persons at the Emergency Department through a Multicomponent Frailty Intervention.” *Age Ageing* (England) 51, no. 2 (2022). <https://doi.org/10.1093/ageing/afab251>.

Chor WPD, Yong PXL, Lim LL, Chai CY, Sim TB, and Kuan WS. “Management of Dyspepsia-The Role of the ED Observation Unit to Optimize Patient Outcomes.” *Am J Emerg Med* (United States) 36, no. 10 (2018): 1733–37. <https://doi.org/10.1016/j.ajem.2018.01.057>.

Claeys KC, Lagnf AM, Patel TB, Jacob MG, Davis SL, and Rybak MJ. “Acute Bacterial Skin and Skin Structure Infections Treated with Intravenous Antibiotics in the Emergency Department or Observational Unit: Experience at the Detroit Medical Center.” *Infect Dis Ther* (New Zealand) 4, no. 2 (2015): 173–86. <https://doi.org/10.1007/s40121-015-0069-7>.

Clery MJ, Olsen E, Marcovitch H, et al. “Safe Discharge Needs Following Emergency Care for Intimate Partner Violence, Sexual Assault, and Sex Trafficking.” *West J Emerg Med* (United States) 24, no. 3 (2023): 615–21. <https://doi.org/10.5811/westjem.59072>.

Cline DM, Silva S, Freiermuth CE, Thornton V, and Tanabe P. “Emergency Department (ED), ED Observation, Day Hospital, and Hospital Admissions for Adults with Sickle Cell Disease.” *West J Emerg Med* (United States) 19, no. 2 (2018): 311–18. <https://doi.org/10.5811/westjem.2017.9.35422>.

Collins SP, Lindsell CJ, Naftilan AJ, et al. “Low-Risk Acute Heart Failure Patients: External Validation of the Society of Chest Pain Center’s Recommendations.” *Crit Pathw Cardiol* (United States) 8, no. 3 (2009): 99–103. <https://doi.org/10.1097/HPC.0b013e3181b5a534>.

Cooper JG, Hammond-Jones D, O’Neill E, et al. “The Clinical Decision Unit Has a Role to Play in the Management of Acute Undifferentiated Abdominal Pain.” *Eur J Emerg Med* (England) 19, no. 5 (2012): 323–28. <https://doi.org/10.1097/MEJ.0b013e32834da85d>.

Cotarlan V, Ho D, Pineda J, Qureshi A, and Shirani J. “Impact of Clinical Predictors and Routine Coronary Artery Disease Testing on Outcome of Patients Admitted to Chest Pain Decision Unit.” *Clin Cardiol* (United States) 37, no. 3 (2014): 146–51. <https://doi.org/10.1002/clc.22229>.

Crenshaw LA, Lindsell CJ, Storrow AB, and Lyons MS. “An Evaluation of Emergency Physician Selection of Observation Unit Patients.” *Am J Emerg Med* (United States) 24, no. 3 (2006): 271–79. <https://doi.org/10.1016/j.ajem.2005.11.002>.

Crilly CJ, Allen AJ, Amato TM, Tiberio A, Schulman RC, and Silverman RA. “Evaluating the Emergency Department Observation Unit for the Management of Hyperglycemia in Adults.” *Am J Emerg Med* (United States) 36, no. 11 (2018): 1975–79. <https://doi.org/10.1016/j.ajem.2018.02.027>.

Cristoni L, Tampieri A, Mucci F, et al. “Cardioversion of Acute Atrial Fibrillation in the Short Observation Unit: Comparison of a Protocol Focused on Electrical Cardioversion with Simple Antiarrhythmic Treatment.” *Emerg Med J* (England) 28, no. 11 (2011): 932–37. <https://doi.org/10.1136/emj.2009.083196>.

Davis A, Chiu J, Lau SK, Kok YJ, and Wu JYH. “Efficacy of Implementation of a Chest Pain Center at a Community Hospital.” *Crit Pathw Cardiol* (United States) 16, no. 4 (2017): 135–41. <https://doi.org/10.1097/HPC.0000000000000133>.

Dawson M, Youngquist S, Bledsoe J, et al. “Low-Risk Young Adult Patients with Chest Pain May Not Benefit from Routine Cardiac Stress Testing: A Bayesian Analysis.” *Crit Pathw Cardiol* (United States) 9, no. 3 (2010): 170–73. <https://doi.org/10.1097/HPC.0b013e3181e6830c>.

Decker WW, Smars PA, Vaidyanathan L, et al. “A Prospective, Randomized Trial of an Emergency Department Observation Unit for Acute Onset Atrial Fibrillation.” *Ann Emerg Med* (United States) 52, no. 4 (2008): 322–28. <https://doi.org/10.1016/j.annemergmed.2007.12.015>.

Dickfos M, Ibrahim H, Evans A, and Franz R. “Cohort Study on Emergency General Surgery Patients and an Observation Unit.” *ANZ J Surg* (Australia) 88, nos. 7–8 (2018): 713–17. <https://doi.org/10.1111/ans.13960>.

Diercks DB, Kirk JD, Turnipseed SD, and Amsterdam EA. “Evaluation of Patients with Methamphetamine- and Cocaine-Related Chest Pain in a Chest Pain Observation Unit.” *Crit Pathw Cardiol* (United States) 6, no. 4 (2007): 161–64. <https://doi.org/10.1097/HPC.0b013e31815991f9>.

Diercks DB, Peacock WF, Kirk JD, and Weber JE. “ED Patients with Heart Failure: Identification of an Observational Unit-Appropriate Cohort.” *Am J Emerg Med* (United States) 24, no. 3 (2006): 319–24. <https://doi.org/10.1016/j.ajem.2005.11.014>.

Dorsey ST, Harrington ET, Iv WF, and Emerman CL. “Ileus and Small Bowel Obstruction in an Emergency Department Observation Unit: Are There Outcome Predictors?” *West J Emerg Med* (United States) 12, no. 4 (2011): 404–7. <https://doi.org/10.5811/westjem.2011.3.2175>.

Drago F, Gasparini G, Signori A, Campisi C, Cozzani E, and Parodi A. “Dermatological Consultations in an Observation Unit of an Emergency Department in Italy.” *J Eur Acad Dermatol Venereol* (England) 29, no. 5 (2015): 973–80. <https://doi.org/10.1111/jdv.12744>.

Dunkley CA, Carpenter JE, Murray BP, et al. “Retrospective Review of a Novel Approach to Buprenorphine Induction in the Emergency Department.” *J Emerg Med* (United States) 57, no. 2 (2019): 181–86. <https://doi.org/10.1016/j.jemermed.2019.03.029>.

Ely S, Chandra A, Mani G, Drake W, Freeman D, and Limkakeng AT Jr. “Utility of Observation Units for Young Emergency Department Chest Pain Patients.” *J Emerg Med* (United States) 44, no. 2 (2013): 306–12. <https://doi.org/10.1016/j.jemermed.2012.07.048>.

Ernst AA, Jones J, Weiss SJ, and Silva O. “Emergency Department Orthopedics Observation Unit as an Alternative to Admission.” *South Med J* (United States) 107, no. 10 (2014): 648–53. <https://doi.org/10.14423/SMJ.0000000000000181>.

Finefrock S. “Nuclear Stress Testing in an Emergency Department Observation Unit.” *J Emerg Nurs* (United States) 32, no. 5 (2006): 420–23. <https://doi.org/10.1016/j.jen.2006.05.026>.

Fish-Trotter H, Collins SP, Danagoulian S, et al. “Design and Rationale of a Randomized Trial: Using Short Stay Units Instead of Routine Admission to Improve Patient Centered Health Outcomes for Acute Heart Failure Patients (SSU-AHF).” *Contemp Clin Trials* (United States) 72 (2018): 137–45. <https://doi.org/10.1016/j.cct.2018.08.003>.

Foo CL, Siu VW, Tan TL, Ding YY, and Seow E. “Geriatric Assessment and Intervention in an Emergency Department Observation Unit Reduced Re-Attendance and Hospitalisation Rates.” *Australas J Ageing* (Australia) 31, no. 1 (2012): 40–46. <https://doi.org/10.1111/j.1741-6612.2010.00499.x>.

Francis E, Marchand W, Hart M, et al. “Utilization and Outcome in an Overnight Psychiatric Observation Program at a Veterans Affairs Medical Center.” *Psychiatr Serv* (United States) 51, no. 1 (2000): 92–95. <https://doi.org/10.1176/ps.51.1.92>.

Fuller M, Hamilton D, Holly J, et al. “Prospective Evaluation of a Simplified Risk Stratification Tool for Patients with Chest Pain in an Emergency Department Observation Unit.” *Crit Pathw Cardiol* (United States) 12, no. 3 (2013): 132–36. <https://doi.org/10.1097/HPC.0b013e31829a79cd>.

Glover M, Gottumukkala RV, Sanchez Y, et al. “Appropriateness of Extremity Magnetic Resonance Imaging Examinations in an Academic Emergency Department Observation Unit.” *West J Emerg Med* (United States) 19, no. 3 (2018): 467–73. <https://doi.org/10.5811/westjem.2018.3.35463>.

Goldsmith LP, Smith JG, Clarke G, et al. “What Is the Impact of Psychiatric Decision Units on Mental Health Crisis Care Pathways? Protocol for an Interrupted Time Series Analysis with a Synthetic Control Study.” *BMC Psychiatry* (England) 20, no. 1 (2020): 185. ISRCTN/ISRCTN77588384. <https://doi.org/10.1186/s12888-020-02581-5>.

Gonnah R, Hegazi MO, Hmdy I, and Shenoda MM. “Can a Change in Policy Reduce Emergency Hospital Admissions? Effect of Admission Avoidance Team, Guideline Implementation and Maximising the Observation Unit.” *Emerg Med J* (England) 25, no. 9 (2008): 575–78. <https://doi.org/10.1136/emj.2007.053090>.

Goodacre S, Mason S, Arnold J, and Angelini K. “Psychologic Morbidity and Health-Related Quality of Life of Patients Assessed in a Chest Pain Observation Unit.” *Ann Emerg Med* (United States) 38, no. 4 (2001): 369–76. <https://doi.org/10.1067/mem.2001.118010>.

Goodacre S, Morris F, Arnold J, and Angelini K. “Is a Chest Pain Observation Unit Likely to Be Cost Saving in a British Hospital?” *Emerg Med J* (England) 18, no. 1 (2001): 11–14. <https://doi.org/10.1136/emj.18.1.11>.

Goodacre S, Nicholl J, Dixon S, et al. “Randomised Controlled Trial and Economic Evaluation of a Chest Pain Observation Unit Compared with Routine Care.” *BMJ* (England) 328, no. 7434 (2004): 254. <https://doi.org/10.1136/bmj.37956.664236.EE>.

Goodacre SW. “Should We Establish Chest Pain Observation Units in the UK? A Systematic Review and Critical Appraisal of the Literature.” *J Accid Emerg Med* (England) 17, no. 1 (2000): 1–6. <https://doi.org/10.1136/emj.17.1.1>.

Goodacre SW, Morris FM, Campbell S, Arnold J, and Angelini K. “A Prospective, Observational Study of a Chest Pain Observation Unit in a British Hospital.” *Emerg Med J* (England) 19, no. 2 (2002): 117–21. <https://doi.org/10.1136/emj.19.2.117>.

Gotz VN, Thompson A, and Jones K. “Developing and Evaluating Nurse Led Discharge in Acute Medicine.” *Acute Med* (England) 13, no. 4 (2014): 159–62.

Grossman AM, Volz KA, Shapiro NI, et al. “Comparison of 1-Day Emergency Department Observation and Inpatient Ward for 1-Day Admissions in Syncope Patients.” *J Emerg Med* (United States) 50, no. 2 (2016): 217–22. <https://doi.org/10.1016/j.jemermed.2015.06.013>.

Grossman SA, Shapiro NI, Mottley JL, Sanchez L, Ullman E, and Wolfe RE. “Is Telemetry Useful in Evaluating Chest Pain Patients in an Observation Unit?” *Intern Emerg Med* (Italy) 6, no. 6 (2011): 543–46. <https://doi.org/10.1007/s11739-011-0648-x>.

Guirgis FW, Gray-Eurom K, Mayfield TL, et al. “Impact of an Abbreviated Cardiac Enzyme Protocol to Aid Rapid Discharge of Patients with Cocaine-Associated Chest Pain in the Clinical Decision Unit.” *West J Emerg Med* (United States) 15, no. 2 (2014): 180–83. <https://doi.org/10.5811/westjem.2013.11.19232>.

Guisado-Gil AB, Mejías-Trueba M, Peñalva G, et al. “Antimicrobial Stewardship in the Emergency Department Observation Unit: Definition of a New Indicator and Evaluation of Antimicrobial Use and Clinical Outcomes.” *Antibiotics (Basel)* (Switzerland) 13, no. 4 (2024). <https://doi.org/10.3390/antibiotics13040356>.

Hahn B, Sunny S, Kettyle P, Chacko J, and Stefanov DG. “Characteristics of Patients Who Return to the Emergency Department after an Observation-Unit Assessment.” *Clin Exp Emerg Med* (Korea (South)) 11, no. 4 (2024): 349–57. <https://doi.org/10.15441/ceem.24.192>.

Hall ME, Miller CD, and Hundley WG. “Adenosine Stress Cardiovascular Magnetic Resonance-Observation Unit Management of Patients at Intermediate Risk for Acute Coronary Syndrome: A Possible Strategy for Reducing Healthcare-Related Costs.” *Curr Treat Options Cardiovasc Med* (United States) 14, no. 1 (2012): 117–25. <https://doi.org/10.1007/s11936-011-0156-3>.

Haran JP, Wu G, Bucci V, et al. “Antibiotic-Associated Diarrhoea in Emergency Department Observation Unit Patients.” *Epidemiol Infect* (England) 144, no. 10 (2016): 2176–83. <https://doi.org/10.1017/S0950268816000200>.

Haran JP, Wu G, Bucci V, Fischer A, Boyer EW, and Hibberd PL. “Treatment of Bacterial Skin Infections in ED Observation Units: Factors Influencing Prescribing Practice.” *Am J Emerg Med* (United States) 33, no. 12 (2015): 1780–85. <https://doi.org/10.1016/j.ajem.2015.08.035>.

Harmouche E, Mahmoud GA, Ross M, Hockenberry J, Dharia R, and Nahab F. “Early Echocardiography Has a Low Yield in Patients with Transient Ischemic Attack.” *J Stroke Cerebrovasc Dis* (United States) 26, no. 8 (2017): 1858–63. <https://doi.org/10.1016/j.jstrokecerebrovasdis.2017.04.022>.

Hartsell S, Dorais J, Preston R, et al. “False-Positive Rates of Provocative Cardiac Testing in Chest Pain Patients Admitted to an Emergency Department Observation Unit.” *Crit Pathw Cardiol* (United States) 13, no. 3 (2014): 104–8. <https://doi.org/10.1097/HPC.0000000000000018>.

Hayden EM, Dorner SC, Sonis JD, and White BA. “Patient Experience With an In-Home COVID Virtual Observation Unit: An Analysis.” *J Patient Exp* (United States) 10 (2023): 23743735231171124. <https://doi.org/10.1177/23743735231171124>.

Hayden EM, Grabowski BG, Kishen EB, Zachrison KS, and White BA. “The Value of an Emergency Medicine Virtual Observation Unit.” *Ann Emerg Med* (United States) 84, no. 3 (2024): 261–69. <https://doi.org/10.1016/j.annemergmed.2024.02.001>.

Helton B, MacWhinnie A, Minor SB, Lodise TP, Rafferty KD, and Allison SL. “Early Directed Oritavancin Therapy in the Emergency Department May Lead to Hospital Avoidance Compared to Standard Treatment for Acute Bacterial Skin and Skin Structure Infections: A Real-World Retrospective Analysis.” *Drugs Real World Outcomes* (Switzerland) 7, no. Suppl 1 (2020): 20–29. <https://doi.org/10.1007/s40801-020-00201-y>.

Holly J, Bledsoe J, Black K, et al. “Prospective Evaluation of an ED Observation Unit Protocol for Trauma Activation Patients.” *Am J Emerg Med* (United States) 30, no. 8 (2012): 1402–6. <https://doi.org/10.1016/j.ajem.2011.10.012>.

Holly J, Fuller M, Hamilton D, et al. “Prospective Evaluation of the Use of the Thrombolysis in Myocardial Infarction Score as a Risk Stratification Tool for Chest Pain Patients Admitted to an ED Observation Unit.” *Am J Emerg Med* (United States) 31, no. 1 (2013): 185–89. <https://doi.org/10.1016/j.ajem.2012.07.006>.

Holly J, Hamilton D, Bledsoe J, et al. “Prospective Evaluation of the Treatment of Intermediate-Risk Chest Pain Patients in an Emergency Department Observation Unit.” *Crit Pathw Cardiol* (United States) 11, no. 1 (2012): 10–13. <https://doi.org/10.1097/HPC.0b013e31824265a8>.

Hostetler B, Leikin JB, Timmons JA, Hanashiro PK, and Kissane K. “Patterns of Use of an Emergency Department-Based Observation Unit.” *Am J Ther* (United States) 9, no. 6 (2002): 499–502. <https://doi.org/10.1097/00045391-200211000-00007>.

Husain I, Mahler SA, Hiestand BC, Miller CD, and Stopyra JP. “The Impact of Accelerated Diagnostic Protocol Implementation on Chest Pain Observation Unit Utilization.” *Crit Pathw Cardiol* (United States) 21, no. 1 (2022): 7–10. ClinicalTrials.gov/NCT02056964. <https://doi.org/10.1097/HPC.0000000000000254>.

Iannone P and Lenzi T. “Effectiveness of a Multipurpose Observation Unit: Before and after Study.” *Emerg Med J* (England) 26, no. 6 (2009): 407–14. <https://doi.org/10.1136/emj.2007.057539>.

Ibrahim I, Macatangay R, Chai CY, Khoo CM, and Mahadevan M. “24-Hr Observation Unit Is Safe Location for Rapid Glucose Control in Uncomplicated Severe Hyperglycaemia.” *BMC Emerg Med* (England) 21, no. 1 (2021): 66. <https://doi.org/10.1186/s12873-021-00460-0>.

Ismail MF, Doherty K, Bradshaw P, O’Sullivan I, and Cassidy EM. “Symptom-Triggered Therapy for Assessment and Management of Alcohol Withdrawal Syndrome in the Emergency Department Short-Stay Clinical Decision Unit.” *Emerg Med J* (England) 36, no. 1 (2019): 18–21. <https://doi.org/10.1136/emermed-2017-206997>.

Jaffe AS. “Use of Biomarkers in the Emergency Department and Chest Pain Unit.” *Cardiol Clin* (Netherlands) 23, no. 4 (2005): 453–65, vi. <https://doi.org/10.1016/j.ccl.2005.08.013>.

Jasani G, Papas M, Patel AJ, et al. “Immediate Stress Echocardiography for Low-Risk Chest Pain Patients in the Emergency Department: A Prospective Observational Cohort Study.” *J Emerg Med* (United States) 54, no. 2 (2018): 156–64. <https://doi.org/10.1016/j.jemermed.2017.10.019>.

Jibrin I, Hamirani YS, Mitikiri N, Ozdegirmenci H, Wentz C, and Bahr RD. “Maryland’s First Inpatient Chest Pain Short Stay Unit as an Alternative to Emergency Room-Based Observation Unit.” *Crit Pathw Cardiol* (United States) 7, no. 1 (2008): 35–42. <https://doi.org/10.1097/HPC.0b013e318163eb83>.

Jones AE, Kennedy M, Hayden EM, et al. “A Protocol to Determine the Acceptability and Feasibility of a Pilot Intervention Emergency Department Virtual Observation Unit Fall Prevention Program.” *Pilot Feasibility Stud* (England) 10, no. 1 (2024): 79. <https://doi.org/10.1186/s40814-024-01502-7>.

Jung OS, Graetz I, Dorner SC, and Hayden EM. “Implementing a COVID-19 Virtual Observation Unit in Emergency Medicine: Frontline Clinician and Staff Experiences.” *Med Care Res Rev* (United States) 80, no. 1 (2023): 79–91. <https://doi.org/10.1177/10775587221108750>.

Katz DA, Graber M, Birrer E, et al. “Health Beliefs toward Cardiovascular Risk Reduction in Patients Admitted to Chest Pain Observation Units.” *Acad Emerg Med* (United States) 16, no. 5 (2009): 379–87. <https://doi.org/10.1111/j.1553-2712.2009.00383.x>.

Katz DA, Graber M, Lounsbury P, et al. “Multiple Risk Factor Counseling to Promote Heart-Healthy Lifestyles in the Chest Pain Observation Unit: Pilot Randomized Controlled Trial.” *Acad Emerg Med* (United States) 24, no. 8 (2017): 968–82. <https://doi.org/10.1111/acem.13231>.

Kendall JL, Kestler AM, Whitaker KT, Adkisson MM, and Haukoos JS. “Blunt Abdominal Trauma Patients Are at Very Low Risk for Intra-Abdominal Injury after Emergency Department Observation.” *West J Emerg Med* (United States) 12, no. 4 (2011): 496–504. <https://doi.org/10.5811/westjem.2010.11.2016>.

Khare RK, Courtney DM, Powell ES, Venkatesh AK, and Lee TA. “Sixty-Four-Slice Computed Tomography of the Coronary Arteries: Cost-Effectiveness Analysis of Patients Presenting to the Emergency Department with Low-Risk Chest Pain.” *Acad Emerg Med* (United States) 15, no. 7 (2008): 623–32. <https://doi.org/10.1111/j.1553-2712.2008.00161.x>.

Khare RK, Powell ES, Venkatesh AK, and Courtney DM. “Diagnostic Uncertainty and Costs Associated with Current Emergency Department Evaluation of Low Risk Chest Pain.” *Crit Pathw Cardiol* (United States) 7, no. 3 (2008): 191–96. <https://doi.org/10.1097/HPC.0b013e318176faa1>.

Khoo KSM, Lim ZY, Chai CY, Mahadevan M, and Kuan WS. “Management of Acute Pyelonephritis in the Emergency Department Observation Unit.” *Singapore Med J* (India) 62, no. 6 (2021): 287–95. <https://doi.org/10.11622/smedj.2020020>.

Kilaru AS, Porges SB, Grossman L, et al. “An Accelerated Hospital Observation Pathway to Reduce Length of Stay for Patients with COVID-19.” *Am J Manag Care* (United States) 28, no. 6 (2022): 262–68. <https://doi.org/10.37765/ajmc.2022.88789>.

Klotz AD, Caterino JM, Durham D, et al. “Observation Unit Use among Patients with Cancer Following Emergency Department Visits: Results of a Multicenter Prospective Cohort from CONCERN.” *Acad Emerg Med* (United States) 29, no. 2 (2022): 174–83. <https://doi.org/10.1111/acem.14392>.

Koenig BO, Ross MA, and Jackson RE. “An Emergency Department Observation Unit Protocol for Acute-Onset Atrial Fibrillation Is Feasible.” *Ann Emerg Med* (United States) 39, no. 4 (2002): 374–81. <https://doi.org/10.1067/mem.2002.122785>.

Kothari D, Babineau M, Hall M, Freedman SD, Shapiro NI, and Sheth SG. “Preventing Hospitalization in Mild Acute Pancreatitis Using a Clinical Pathway in the Emergency Department.” *J Clin Gastroenterol* (United States) 52, no. 8 (2018): 734–41. <https://doi.org/10.1097/MCG.0000000000000954>.

Krantz MJ, Zwang O, Rowan SB, et al. “A Cooperative Care Model: Cardiologists and Hospitalists Reduce Length of Stay in a Chest Pain Observation Unit.” *Crit Pathw Cardiol* (United States) 4, no. 2 (2005): 55–58. <https://doi.org/10.1097/01.hpc.0000164083.44679.75>.

Krishnamani PP, Qdaisat A, Wattana MK, et al. “Characteristics and Outcomes of Patients with Cancer Pain Placed in an Emergency Department Observation Unit.” *Cancers (Basel)* (Switzerland) 14, no. 23 (2022). <https://doi.org/10.3390/cancers14235871>.

Krishnamani PP, Sandoval M, Chaftari P, et al. “The Value Proposition of Observation Medicine in Managing Acute Oncologic Pain.” *Curr Oncol Rep* (United States) 24, no. 5 (2022): 595–602. <https://doi.org/10.1007/s11912-022-01245-9>.

Kuan KK, Lim HC, Goh G, et al. “Cost Savings and Efficacy in Management of Paracetamol Poisoning in a 23-Hours Emergency Department Observation Unit: A Comparison to Inpatient Care.” *Cureus* (United States) 11, no. 12 (2019): e6294. <https://doi.org/10.7759/cureus.6294>.

Kuan WS, Lather KS, and Mahadevan M. “Primary Spontaneous Pneumothorax--the Role of the Emergency Observation Unit.” *Am J Emerg Med* (United States) 29, no. 3 (2011): 293–98. <https://doi.org/10.1016/j.ajem.2009.11.005>.

Lai C, Noeller TP, Schmidt K, King P, and Emerman CL. “Short-Term Risk after Initial Observation for Chest Pain.” *J Emerg Med* (United States) 25, no. 4 (2003): 357–62. <https://doi.org/10.1016/s0736-4679(03)00238-5>.

Lauritano EC, Novi A, Santoro MC, and Casagranda I. “Incidence, Clinical Features and Management of Acute Allergic Reactions: The Experience of a Single, Italian Emergency Department.” *Eur Rev Med Pharmacol Sci* (Italy) 17 Suppl 1 (2013): 39–44.

Leykum LK, Huerta V, and Mortensen E. “Implementation of a Hospitalist-Run Observation Unit and Impact on Length of Stay (LOS): A Brief Report.” *J Hosp Med* (United States) 5, no. 9 (2010): E2-5. <https://doi.org/10.1002/jhm.642>.

Limkakeng AT, Glickman SW, Cairns CB, and Chandra A. “Unsuspected Pulmonary Embolism in Observation Unit Patients.” *West J Emerg Med* (United States) 10, no. 3 (2009): 130–34.

Limkakeng AT Jr and Chandra A. “Impact of Renal Dysfunction on Acute Coronary Syndrome Evaluation in Observation Unit Patients.” *Am J Emerg Med* (United States) 28, no. 6 (2010): 658–62. <https://doi.org/10.1016/j.ajem.2009.02.014>.

Lin Z, Lim SH, Yap QV, et al. “Comparing Conventional and High Sensitivity Troponin T Measurements in Identifying Adverse Cardiac Events in Patients Admitted to an Asian Emergency Department Chest Pain Observation Unit.” *Int J Cardiol Heart Vasc* (Ireland) 34 (2021): 100758. <https://doi.org/10.1016/j.ijcha.2021.100758>.

Lipitz-Snyderman A, Klotz A, Gennarelli RL, and Groeger J. “A Population-Based Assessment of Emergency Department Observation Status for Older Adults With Cancer.” *J Natl Compr Canc Netw* (United States) 15, no. 10 (2017): 1234–39. <https://doi.org/10.6004/jnccn.2017.0160>.

Liu SW, Thatphet P, Wongtangman T, et al. “Predicting Falls with Ultrasound, Physical Parameters or Fall-Risk Questions among Older Adults: A Prospective Cohort Study.” *Am J Emerg Med* (United States) 91 (2025): 132–38. <https://doi.org/10.1016/j.ajem.2024.10.008>.

Llanos-Torres KH, Pérez-Orozco R, and Málaga G. “Nosocomial Infections in Emergency Observation Units and Their Association with Overcrowding and Ventilation.” *Rev Peru Med Exp Salud Publica* (Peru) 37, no. 4 (2020): 721–25. <https://doi.org/10.17843/rpmesp.2020.374.5192>.

Lygrisse KA, Zak S, Singh V, Hutzler LH, Schwarzkopf R, and Rozell JC. “Emergency Department Observation Versus Readmission Following Total Joint Arthroplasty: Can We Avoid the Bundle Buster?” *J Arthroplasty* (United States) 36, no. 3 (2021): 833–36. <https://doi.org/10.1016/j.arth.2020.09.021>.

Lyon M, Sturgis L, Lottenberg R, et al. “Outcomes of an Emergency Department Observation Unit-Based Pathway for the Treatment of Uncomplicated Vaso-Occlusive Events in Sickle Cell Disease.” *Ann Emerg Med* (United States) 76, no. 3S (2020): S12–20. <https://doi.org/10.1016/j.annemergmed.2020.08.007>.

Madsen T, Bledsoe J, and Bossart P. “Physician Documentation of Nonspecific EKG Changes Predicts Hospital Admission among Observation Unit Chest Pain Patients.” *Crit Pathw Cardiol* (United States) 8, no. 1 (2009): 34–37. <https://doi.org/10.1097/HPC.0b013e3181978fc0>.

Madsen T, Bossart P, Bledsoe J, et al. “Patients with Coronary Disease Fail Observation Status at Higher Rates than Patients without Coronary Disease.” *Am J Emerg Med* (United States) 28, no. 1 (2010): 19–22. <https://doi.org/10.1016/j.ajem.2008.09.021>.

Madsen T, Dawson M, Bledsoe J, and Bossart P. “Serial Hematocrit Testing Does Not Identify Major Injuries in Trauma Patients in an Observation Unit.” *Am J Emerg Med* (United States) 28, no. 4 (2010): 472–76. <https://doi.org/10.1016/j.ajem.2009.01.034>.

Madsen T, Mallin M, Bledsoe J, et al. “Utility of the Emergency Department Observation Unit in Ensuring Stress Testing in Low-Risk Chest Pain Patients.” *Crit Pathw Cardiol* (United States) 8, no. 3 (2009): 122–24. <https://doi.org/10.1097/HPC.0b013e3181b00782>.

Madsen T, Perkins R, Holt B, et al. “Emergency Department Observation Unit Utilization Among Older Patients With Chest Pain.” *Crit Pathw Cardiol* (United States) 18, no. 1 (2019): 19–22. <https://doi.org/10.1097/HPC.0000000000000166>.

Madsen T, Smyres C, Wood T, et al. “Cardiology Consultation Reduces Provocative Testing Rates in an ED Observation Unit.” *Am J Emerg Med* (United States) 35, no. 1 (2017): 25–28. <https://doi.org/10.1016/j.ajem.2016.09.032>.

Madsen TE, Bledsoe J, and Bossart P. “Appropriately Screened Geriatric Chest Pain Patients in an Observation Unit Are Not Admitted at a Higher Rate than Nongeriatric Patients.” *Crit Pathw Cardiol* (United States) 7, no. 4 (2008): 245–47. <https://doi.org/10.1097/HPC.0b013e31818efb86>.

Madsen TE, Bledsoe JR, and Bossart PJ. “Observation Unit Admission as an Alternative to Inpatient Admission for Trauma Activation Patients.” *Emerg Med J* (England) 26, no. 6 (2009): 421–23. <https://doi.org/10.1136/emj.2008.064626>.

Madsen TE, Fuller M, Hartsell S, Hamilton D, and Bledsoe J. “Prospective Evaluation of Outcomes among Geriatric Chest Pain Patients in an ED Observation Unit.” *Am J Emerg Med* (United States) 34, no. 2 (2016): 207–11. <https://doi.org/10.1016/j.ajem.2015.10.010>.

Madsen TE, Stewart M, Smyres C, et al. “Significance of an Indeterminate Troponin I in Patients Evaluated for Chest Pain in an Emergency Department Observation Unit.” *Crit Pathw Cardiol* (United States) 14, no. 4 (2015): 146–49. <https://doi.org/10.1097/HPC.0000000000000054>.

Mahler SA, Hiestand BC, Nwanaji-Enwerem J, et al. “Reduction in Observation Unit Length of Stay with Coronary Computed Tomography Angiography Depends on Time of Emergency Department Presentation.” *Acad Emerg Med* (United States) 20, no. 3 (2013): 231–39. <https://doi.org/10.1111/acem.12094>.

Mahony CR, Traynor MD Jr, Knight AW, et al. “Small Bowel Obstruction Managed without Hospital Admission: A Safe Way to Reduce Both Cost and Time in the Hospital?” *Surgery* (United States) 171, no. 6 (2022): 1665–70. <https://doi.org/10.1016/j.surg.2021.10.041>.

Manoli A 3rd, Markel JF, Pizzimenti NM, and Markel DC. “Use of a Mandatory Clinical Decision Unit Reduces Readmission Rates Following Total Joint Arthroplasty.” *J Knee Surg* (Germany) 34, no. 9 (2021): 924–29. <https://doi.org/10.1055/s-0039-3402053>.

Marshall JR, Katzer R, Lotfipour S, et al. “Use of Physician-in-Triage Model in the Management of Abdominal Pain in an Emergency Department Observation Unit.” *West J Emerg Med* (United States) 18, no. 2 (2017): 181–88. <https://doi.org/10.5811/westjem.2016.10.32042>.

Martinez E, Reilly BM, Evans AT, and Roberts RR. “The Observation Unit: A New Interface between Inpatient and Outpatient Care.” *Am J Med* (United States) 110, no. 4 (2001): 274–77. <https://doi.org/10.1016/s0002-9343(00)00710-5>.

Mechanic OJ, Pascheles CY, Lopez GJ, et al. “Using the Boston Syncope Observation Management Pathway to Reduce Hospital Admission and Adverse Outcomes.” *West J Emerg Med* (United States) 20, no. 2 (2019): 250–55. <https://doi.org/10.5811/westjem.2018.11.39657>.

Meldon S, Saxena S, Hashmi A, et al. “Impact of Geriatric Consult Evaluations on Hospital Admission Rates for Older Adults.” *West J Emerg Med* (United States) 25, no. 1 (2024): 86–93. <https://doi.org/10.5811/westjem.60664>.

Menditto VG, Gabrielli B, Marcosignori M, et al. “A Management of Blunt Thoracic Trauma in an Emergency Department Observation Unit: Pre-Post Observational Study.” *J Trauma Acute Care Surg* (United States) 72, no. 1 (2012): 222–28. <https://doi.org/10.1097/TA.0b013e3182140cad>.

Menditto VG, Lucci M, Polonara S, Pomponio G, and Gabrielli A. “Management of Minor Head Injury in Patients Receiving Oral Anticoagulant Therapy: A Prospective Study of a 24-Hour Observation Protocol.” *Ann Emerg Med* (United States) 59, no. 6 (2012): 451–55. <https://doi.org/10.1016/j.annemergmed.2011.12.003>.

Miller CD, Case LD, Little WC, et al. “Stress CMR Reduces Revascularization, Hospital Readmission, and Recurrent Cardiac Testing in Intermediate-Risk Patients with Acute Chest Pain.” *JACC Cardiovasc Imaging* (United States) 6, no. 7 (2013): 785–94. ClinicalTrials.gov/NCT01035047. <https://doi.org/10.1016/j.jcmg.2012.11.022>.

Miller CD, Hwang W, Case D, et al. “Stress CMR Imaging Observation Unit in the Emergency Department Reduces 1-Year Medical Care Costs in Patients with Acute Chest Pain: A Randomized Study for Comparison with Inpatient Care.” *JACC Cardiovasc Imaging* (United States) 4, no. 8 (2011): 862–70. ClinicalTrials.gov/NCT00678639. <https://doi.org/10.1016/j.jcmg.2011.04.016>.

Miller CD, Hwang W, Hoekstra JW, et al. “Stress Cardiac Magnetic Resonance Imaging with Observation Unit Care Reduces Cost for Patients with Emergent Chest Pain: A Randomized Trial.” *Ann Emerg Med* (United States) 56, no. 3 (2010): 209-219.e2. <https://doi.org/10.1016/j.annemergmed.2010.04.009>.

Miller CD, Stopyra JP, Mahler SA, et al. “ACES (Accelerated Chest Pain Evaluation With Stress Imaging) Protocols Eliminate Testing Disparities in Patients With Chest Pain.” *Crit Pathw Cardiol* (United States) 18, no. 1 (2019): 5–9. <https://doi.org/10.1097/HPC.0000000000000161>.

Mong R, Arciaga GJ, and Tan HH. “Use of a 23-Hour Emergency Department Observation Unit for the Management of Patients with Toxic Exposures.” *Emerg Med J* (England) 34, no. 11 (2017): 755–60. <https://doi.org/10.1136/emermed-2016-206531>.

Muhamed S, Konzelmann J, Reed L, and Holstein H. “Evaluating the Impact of Protocol-Driven Treatment for COVID-19 in an Emergency Department Observation Unit.” *Cureus* (United States) 14, no. 9 (2022): e29683. <https://doi.org/10.7759/cureus.29683>.

Muhamed S, Vassy M, Konzelmann J, Gibson J, and Pack L. “Utility of an Emergency Department Observation Unit in Providing Care for Patients With Blunt Thoracic Trauma.” *Cureus* (United States) 15, no. 5 (2023): e39447. <https://doi.org/10.7759/cureus.39447>.

Nahab F, Leach G, Kingston C, et al. “Impact of an Emergency Department Observation Unit Transient Ischemic Attack Protocol on Length of Stay and Cost.” *J Stroke Cerebrovasc Dis* (United States) 21, no. 8 (2012): 673–78. <https://doi.org/10.1016/j.jstrokecerebrovasdis.2011.02.017>.

Napoli AM. “Inter-Rater Reliability of the Diamond & Forrester Score in Emergency Department Chest Pain Observation Unit Patients.” *Crit Pathw Cardiol* (United States) 14, no. 4 (2015): 154–56. <https://doi.org/10.1097/HPC.0000000000000056>.

Napoli AM. “The Association between Pretest Probability of Coronary Artery Disease and Stress Test Utilization and Outcomes in a Chest Pain Observation Unit.” *Acad Emerg Med* (United States) 21, no. 4 (2014): 401–7. <https://doi.org/10.1111/acem.12354>.

Napoli AM, Arrighi JA, Siket MS, and Gibbs FJ. “Physician Discretion Is Safe and May Lower Stress Test Utilization in Emergency Department Chest Pain Unit Patients.” *Crit Pathw Cardiol* (United States) 11, no. 1 (2012): 26–31. <https://doi.org/10.1097/HPC.0b013e3182457bee>.

Napoli AM, Baird J, Tran S, and Wang J. “Low Adverse Event Rates But High Emergency Department Utilization in Chest Pain Patients Treated in an Emergency Department Observation Unit.” *Crit Pathw Cardiol* (United States) 16, no. 1 (2017): 15–21. <https://doi.org/10.1097/HPC.0000000000000099>.

Napoli AM, Tran S, and Wang J. “Low-Risk Chest Pain Patients Younger than 40 Years Do Not Benefit from Admission and Stress Testing.” *Crit Pathw Cardiol* (United States) 12, no. 4 (2013): 201–3. <https://doi.org/10.1097/HPC.0b013e3182a75e3f>.

Navas A, Guzman B, Hassan A, et al. “Untapped Potential for Emergency Department Observation Unit Use: A National Hospital Ambulatory Medical Care Survey (NHAMCS) Study.” *West J Emerg Med* (United States) 23, no. 2 (2022): 134–40. <https://doi.org/10.5811/westjem.2021.8.52231>.

Ng CCC, Kowalski S, Mu WL, et al. “Evaluating Smoking Cessation Service at an Emergency Department Clinical Observation Unit.” *Am J Manag Care* (United States) 28, no. 10 (2022): e388–91. <https://doi.org/10.37765/ajmc.2022.89256>.

Nouvenne A, Ticinesi A, Cerundolo N, et al. “Implementing a Multidisciplinary Rapid Geriatric Observation Unit for Non-Critical Older Patients Referred to Hospital: Observational Study on Real-World Data.” *Aging Clin Exp Res* (Germany) 34, no. 3 (2022): 599–609. <https://doi.org/10.1007/s40520-021-01967-z>.

Numata K, Jones AE, Meeker MA, et al. “An Emergency Department Virtual Observation Unit Fall Prevention Program: A Pilot Acceptability Study.” *Cureus* (United States) 17, no. 2 (2025): e78624. <https://doi.org/10.7759/cureus.78624>.

Numeroso F, Mossini G, Montali F, Lippi G, and Cervellin G. “Prognostic Value of the OESIL Risk Score in a Cohort of Emergency Department Patients with Syncope.” *Minerva Med* (Italy) 104, no. 4 (2013): 413–19.

Numeroso F, Mossini G, Spaggiari E, and Cervellin G. “Syncope in the Emergency Department of a Large Northern Italian Hospital: Incidence, Efficacy of a Short-Stay Observation Ward and Validation of the OESIL Risk Score.” *Emerg Med J* (England) 27, no. 9 (2010): 653–58. <https://doi.org/10.1136/emj.2009.077701>.

Ochoa-Gomez J, Villar-Arias A, Echeverría-Echarri L, Ramall-Gómara E, Ruiz-Azpiazu JI, and Bragado-Blas L. “Attendance of Patients with Minor Head Injury in an Emergency Department Observation Ward.” *Eur J Emerg Med* (England) 7, no. 4 (2000): 267–70. <https://doi.org/10.1097/00063110-200012000-00003>.

Oostema JA, Delano M, Bhatt A, and Brown MD. “Incorporating Diffusion-Weighted Magnetic Resonance Imaging into an Observation Unit Transient Ischemic Attack Pathway: A Prospective Study.” *Neurohospitalist* (United States) 4, no. 2 (2014): 66–73. <https://doi.org/10.1177/1941874413519804>.

Osborne AD, Moore B, Ross MA, and Pitts SR. “The Feasibility of Rubidium-82 Positron Emission Tomography Stress Testing in Low-Risk Chest Pain Protocol Patients.” *Crit Pathw Cardiol* (United States) 10, no. 1 (2011): 41–43. <https://doi.org/10.1097/HPC.0b013e31820d6a2e>.

Patel SK, Elshaboury RH, Gandhi RG, Hayes BD, Yun BJ, and Koehl JL. “Assessment and Optimization of the Empiric Treatment of Urinary Tract Infections in an Academic Emergency Department Observation Unit.” *J Emerg Med* (United States) 58, no. 2 (2020): 203–10. <https://doi.org/10.1016/j.jemermed.2019.12.021>.

Peacock WF 4th, Holland R, Gyarmathy R, et al. “Observation Unit Treatment of Heart Failure with Nesiritide: Results from the Proaction Trial.” *J Emerg Med* (United States) 29, no. 3 (2005): 243–52. <https://doi.org/10.1016/j.jemermed.2005.01.024>.

Peacock WF 4th, Remer EE, Aponte J, Moffa DA, Emerman CE, and Albert NM. “Effective Observation Unit Treatment of Decompensated Heart Failure.” *Congest Heart Fail* (United States) 8, no. 2 (2002): 68–73. <https://doi.org/10.1111/j.1527-5299.2002.01519.x>.

Pena M, Tsao J, Falaiye T, et al. “Why Won’t Emergency Physicians Discharge Patients With a Low HEART Score From an Observation Unit Without Further Evaluation?” *Crit Pathw Cardiol* (United States) 19, no. 4 (2020): 195–99. <https://doi.org/10.1097/HPC.0000000000000236>.

Pena ME, Jakob MR, Cohen GI, et al. “Reduction in Radiation Exposure through a Stress Test Algorithm in an Emergency Department Observation Unit.” *West J Emerg Med* (United States) 17, no. 2 (2016): 97–103. <https://doi.org/10.5811/westjem.2015.12.27895>.

Pena ME, Pearson CL, Goulet MP, et al. “A 90-Second Magnetocardiogram Using a Novel Analysis System to Assess for Coronary Artery Stenosis in Emergency Department Observation Unit Chest Pain Patients.” *Int J Cardiol Heart Vasc* (Ireland) 26 (2020): 100466. <https://doi.org/10.1016/j.ijcha.2019.100466>.

Pervez T and Malik M. “Tertiary Trauma Survey on Emergency Department Observational Units: A Systematic Literature Review.” *Cureus* (United States) 16, no. 1 (2024): e53187. <https://doi.org/10.7759/cureus.53187>.

Plummer L, Sridhar S, Beninato M, and Parlman K. “Physical Therapist Practice in the Emergency Department Observation Unit: Descriptive Study.” *Phys Ther* (United States) 95, no. 2 (2015): 249–56. <https://doi.org/10.2522/ptj.20140017>.

Powell A, Clark P, and Shah K. “Hospital Development of a Hybrid Emergency Department-Inpatient Care Observation Unit.” *J Emerg Nurs* (United States) 49, no. 6 (2023): 853–62. <https://doi.org/10.1016/j.jen.2023.07.002>.

Powell ES, Patterson BW, Venkatesh AK, and Khare RK. “Cost-Effectiveness of a Novel Indication of Computed Tomography of the Coronary Arteries.” *Crit Pathw Cardiol* (United States) 11, no. 1 (2012): 20–25. <https://doi.org/10.1097/HPC.0b013e318246854c>.

Prabhakar AM, Misono AS, Harvey HB, Yun BJ, Saini S, and Oklu R. “Imaging Utilization from the ED: No Difference between Observation and Admitted Patients.” *Am J Emerg Med* (United States) 33, no. 8 (2015): 1076–79. <https://doi.org/10.1016/j.ajem.2015.04.025>.

Pruitt P, Penn J, Peak D, and Borczuk P. “Identifying Patients with Mild Traumatic Intracranial Hemorrhage at Low Risk of Decompensation Who Are Safe for ED Observation.” *Am J Emerg Med* (United States) 35, no. 2 (2017): 255–59. <https://doi.org/10.1016/j.ajem.2016.10.064>.

Reed MJ, Karuranga S, Kearns D, et al. “Management of Syncope in the Emergency Department: A European Prospective Cohort Study (SEED).” *Eur J Emerg Med* (England) 31, no. 2 (2024): 136–46. ClinicalTrials.gov/NCT05571254. <https://doi.org/10.1097/MEJ.0000000000001101>.

Rentala M, Andrews S, Tiberio A, et al. “Intravenous Home Infusion Therapy Instituted From a 24-Hour Clinical Decision Unit For Patients With Cellulitis.” *Am J Emerg Med* (United States) 34, no. 7 (2016): 1273–75. <https://doi.org/10.1016/j.ajem.2016.04.022>.

Robinson DJ, Woods PG, Snedeker CA, Lynch JH, and Chambers K. “A Comparison Trial for Stratifying Intermediate-Risk Chest Pain: Benefits of Emergency Department Observation Centers.” *Prev Cardiol* (United States) 5, no. 1 (2002): 23–30. <https://doi.org/10.1111/j.1520-037x.2002.00550.x>.

Root BK, Kanter JH, Calnan DC, Reyes-Zaragosa M, Gill HS, and Lanter PL. “Emergency Department Observation of Mild Traumatic Brain Injury with Minor Radiographic Findings: Shorter Stays, Less Expensive, and No Increased Risk Compared to Hospital Admission.” *J Am Coll Emerg Physicians Open* (United States) 1, no. 4 (2020): 609–17. <https://doi.org/10.1002/emp2.12124>.

Ross MA, Compton S, Medado P, Fitzgerald M, Kilanowski P, and O’Neil BJ. “An Emergency Department Diagnostic Protocol for Patients with Transient Ischemic Attack: A Randomized Controlled Trial.” *Ann Emerg Med* (United States) 50, no. 2 (2007): 109–19. <https://doi.org/10.1016/j.annemergmed.2007.03.008>.

Ross MA, Davis B, and Dresselhouse A. “The Role of an Emergency Department Observation Unit in a Clinical Pathway for Atrial Fibrillation.” *Crit Pathw Cardiol* (United States) 3, no. 1 (2004): 8–12. <https://doi.org/10.1097/01.hpc.0000116582.64827.2f>.

Ross MA, Naylor S, Compton S, Gibb KA, and Wilson AG. “Maximizing Use of the Emergency Department Observation Unit: A Novel Hybrid Design.” *Ann Emerg Med* (United States) 37, no. 3 (2001): 267–74. <https://doi.org/10.1067/mem.2001.111519>.

Rouhani SA, Marsh RH, Rimpel L, et al. “Protocolized Emergency Department Observation Care Improves Quality of Ischemic Stroke Care in Haiti.” *Afr J Emerg Med* (Netherlands) 10, no. 3 (2020): 145–51. <https://doi.org/10.1016/j.afjem.2020.05.007>.

Russell FM, Wang A, Ehrman RR, Jacobs J, Croft A, and Larsen C. “Risk Factors Associated with Hospital Admission in COVID-19 Patients Initially Admitted to an Observation Unit.” *Am J Emerg Med* (United States) 46 (2021): 339–43. <https://doi.org/10.1016/j.ajem.2020.10.009>.

Savioli G, Ceresa IF, Manzoni F, Ricevuti G, Bressan MA, and Oddone E. “Role of a Brief Intensive Observation Area with a Dedicated Team of Doctors in the Management of Acute Heart Failure Patients: A Retrospective Observational Study.” *Medicina (Kaunas)* (Switzerland) 56, no. 5 (2020). <https://doi.org/10.3390/medicina56050251>.

Schrager J, Wheatley M, Georgiopoulou V, et al. “Favorable Bed Utilization and Readmission Rates for Emergency Department Observation Unit Heart Failure Patients.” *Acad Emerg Med* (United States) 20, no. 6 (2013): 554–61. <https://doi.org/10.1111/acem.12147>.

Schrock JW, Laskey S, and Cydulka RK. “Predicting Observation Unit Treatment Failures in Patients with Skin and Soft Tissue Infections.” *Int J Emerg Med* (England) 1, no. 2 (2008): 85–90. <https://doi.org/10.1007/s12245-008-0029-z>.

Schrock JW, Reznikova S, and Weller S. “The Effect of an Observation Unit on the Rate of ED Admission and Discharge for Pyelonephritis.” *Am J Emerg Med* (United States) 28, no. 6 (2010): 682–88. <https://doi.org/10.1016/j.ajem.2009.03.003>.

Schulman-Rosenbaum RC, Hadzibabic N, Cuan K, et al. “Use of Endocrine Consultation for Hemoglobin A1C ≥9.0% as a Standardized Practice in an Emergency Department Observation Unit.” *Endocr Pract* (United States) 27, no. 11 (2021): 1133–38. <https://doi.org/10.1016/j.eprac.2021.06.018>.

Sengupta R, Loftus TM, Doers M, et al. “Resting Borg Score as a Predictor of Safe Discharge of Chronic Obstructive Pulmonary Disease from the Emergency Department Observation Unit.” *Acad Emerg Med* (United States) 27, no. 12 (2020): 1302–11. <https://doi.org/10.1111/acem.14091>.

Shah PP, Gupta N, Sharma A, et al. “Chest Pain Unit Using Thrombolysis in Myocardial Infarction Score Risk Stratification: An Impact on the Length of Stay and Cost Savings.” *Crit Pathw Cardiol* (United States) 11, no. 4 (2012): 206–10. <https://doi.org/10.1097/HPC.0b013e31826cc254>.

Shastry S, Yeo J, Richardson LD, and Manini AF. “Observation Unit Management of Low-Risk Emergency Department Patients with Acute Drug Overdose.” *Clin Toxicol (Phila)* (England) 58, no. 7 (2020): 773–76. <https://doi.org/10.1080/15563650.2019.1666986>.

Shaw GJ, Meunier JM, Korfhagen J, et al. “Randomized Controlled Noninferiority Trial Comparing Daptomycin to Vancomycin for the Treatment of Complicated Skin and Skin Structure Infections in an Observation Unit.” *J Emerg Med* (United States) 49, no. 6 (2015): 928–36. <https://doi.org/10.1016/j.jemermed.2015.07.026>.

Sherwood K, Sugerman S, Bossart P, et al. “EDOU Staffing by PAs: What Are the Effects on Patient Outcomes?” *JAAPA* (United States) 24, no. 8 (2011): 31–34, 37. <https://doi.org/10.1097/01720610-201108000-00007>.

Singleton JM, Bilello LA, Greige T, et al. “Outcomes of a Novel ED Observation Pathway for Mild Traumatic Brain Injury and Associated Intracranial Hemorrhage.” *Am J Emerg Med* (United States) 45 (2021): 340–44. <https://doi.org/10.1016/j.ajem.2020.08.093>.

Soremekun OA, Hamedani A, Shofer FS, O’Conor KJ, Svenson J, and Hollander JE. “Safety of a Rapid Diagnostic Protocol with Accelerated Stress Testing.” *Am J Emerg Med* (United States) 32, no. 2 (2014): 124–28. <https://doi.org/10.1016/j.ajem.2013.10.020>.

Southerland LT, Alnemer A, Laufenberg C, Nimjee SM, and Bischof JJ. “The Brain Injury Guidelines (BIG) and Emergency Department Observation and Admission Rates: A Retrospective Cohort Study.” *Am J Emerg Med* (United States) 82 (2024): 37–41. <https://doi.org/10.1016/j.ajem.2024.05.004>.

Southerland LT, Simerlink SR, Smith ZJ, and Sharkey-Toppen TP. “A Quality Improvement Initiative to Decrease Hospital Admission for Skin and Soft Tissue Infections From an Observation Unit.” *J Am Coll Emerg Physicians Open* (United States) 6, no. 3 (2025): 100140. <https://doi.org/10.1016/j.acepjo.2025.100140>.

Southerland LT, Simerlink SR, Vargas AJ, et al. “Beyond Observation: Protocols and Capabilities of an Emergency Department Observation Unit.” *Am J Emerg Med* (United States) 37, no. 10 (2019): 1864–70. <https://doi.org/10.1016/j.ajem.2018.12.049>.

Southerland LT, Stephens JA, Carpenter CR, et al. “Study Protocol for IMAGE: Implementing Multidisciplinary Assessments for Geriatric Patients in an Emergency Department Observation Unit, a Hybrid Effectiveness/Implementation Study Using the Consolidated Framework for Implementation Research.” *Implement Sci Commun* (England) 1 (2020): 28. ClinicalTrials.gov/NCT04068311. <https://doi.org/10.1186/s43058-020-00015-7>.

Southerland LT, Vargas AJ, Nagaraj L, Gure TR, and Caterino JM. “An Emergency Department Observation Unit Is a Feasible Setting for Multidisciplinary Geriatric Assessments in Compliance With the Geriatric Emergency Department Guidelines.” *Acad Emerg Med* (United States) 25, no. 1 (2018): 76–82. <https://doi.org/10.1111/acem.13328>.

Stead LG, Bellolio MF, Suravaram S, et al. “Evaluation of Transient Ischemic Attack in an Emergency Department Observation Unit.” *Neurocrit Care* (United States) 10, no. 2 (2009): 204–8. <https://doi.org/10.1007/s12028-008-9146-z>.

Stewart M, Bledsoe J, Madsen T, et al. “Utilization and Safety of a Pulmonary Embolism Treatment Protocol in an Emergency Department Observation Unit.” *Crit Pathw Cardiol* (United States) 14, no. 3 (2015): 87–89. <https://doi.org/10.1097/HPC.0000000000000046>.

Storrow AB, Collins SP, Lyons MS, Wagoner LE, Gibler WB, and Lindsell CJ. “Emergency Department Observation of Heart Failure: Preliminary Analysis of Safety and Cost.” *Congest Heart Fail* (United States) 11, no. 2 (2005): 68–72. <https://doi.org/10.1111/j.1527-5299.2005.03844.x>.

Styron JF, Jois-Bilowich P, Tallman T, Emerman C, Starling RC, and Frank Peacock W. “Outcomes Associated with Nesiritide Administration for Acute Decompensated Heart Failure in the Emergency Department Observation Unit: A Single Center Experience.” *Congest Heart Fail* (United States) 15, no. 3 (2009): 103–7. <https://doi.org/10.1111/j.1751-7133.2009.00065.x>.

Sun BC, McCreath H, Liang LJ, et al. “Randomized Clinical Trial of an Emergency Department Observation Syncope Protocol versus Routine Inpatient Admission.” *Ann Emerg Med* (United States) 64, no. 2 (2014): 167–75. <https://doi.org/10.1016/j.annemergmed.2013.10.029>.

Sun J, D’Souza M, Losak M, Htet N, Miles-Threatt C, and Mitarai T. “ED Observation Unit-Based Delayed Comfort Care Pathway for ED Patients on Life Support.” *Am J Emerg Med* (United States) 90 (2025): 93–97. <https://doi.org/10.1016/j.ajem.2025.01.031>.

Sztajnkrycer MD, Mell HK, and Melin GJ. “Development and Implementation of an Emergency Department Observation Unit Protocol for Deliberate Drug Ingestion in Adults - Preliminary Results.” *Clin Toxicol (Phila)* (England) 45, no. 5 (2007): 499–504. <https://doi.org/10.1080/15563650701354168>.

Tabbut MP, Schrock JW, Emerman CL, Gramer D, and Jones RA. “Carotid Intima-Media Thickening Predicts Negative Stress Test in Chest Pain Patients in an Emergency Department Observation Unit.” *Am J Emerg Med* (United States) 37, no. 7 (2019): 1385–87. <https://doi.org/10.1016/j.ajem.2018.12.053>.

Taha JJ, Hughes GB, Keadey MT, et al. “The Feasibility of Emergency Department Observation Units in the Management of Mild to Moderate Hyponatremia.” *Am J Emerg Med* (United States) 80 (2024): 11–17. <https://doi.org/10.1016/j.ajem.2024.02.037>.

Takakuwa KM, Halpern EJ, and Shofer FS. “A Time and Imaging Cost Analysis of Low-Risk ED Observation Patients: A Conservative 64-Section Computed Tomography Coronary Angiography ‘Triple Rule-out’ Compared to Nuclear Stress Test Strategy.” *Am J Emerg Med* (United States) 29, no. 2 (2011): 187–95. <https://doi.org/10.1016/j.ajem.2009.09.002>.

Tang D, Chan WL, and Phua DH. “Performance of an Emergency Department Observation Unit Protocol in Reducing Length of Stay for Acetaminophen Overdose: A Retrospective Study.” *Int J Emerg Med* (England) 11, no. 1 (2018): 48. <https://doi.org/10.1186/s12245-018-0210-y>.

Thiruvengadam N, Anderson KL, and Sheth SG. “Significant Projected Savings with Expansion of an Emergency Department Observation Protocol for Mild Acute Pancreatitis.” *Pancreatology* (Switzerland) 25, no. 1 (2025): 35–38. <https://doi.org/10.1016/j.pan.2024.12.009>.

Tijunelis MA, Hanashiro P, Kissane K, Leikin JB, Timmons JA, and Hryhorczuk DO. “Observation Unit Evaluation of Low Risk Drug-Related Chest Pain.” *Am J Emerg Med* (United States) 19, no. 6 (2001): 533–34. <https://doi.org/10.1053/ajem.2001.27168>.

Tran T, Imperato N, Dym A, et al. “The Promising Use of an Emergency Department Observation Unit to Manage Patients with Opioid Use Disorder.” *Am J Emerg Med* (United States) 92 (2025): 152–55. <https://doi.org/10.1016/j.ajem.2025.02.007>.

Ungar A, Tesi F, Chisciotti VM, et al. “Assessment of a Structured Management Pathway for Patients Referred to the Emergency Department for Syncope: Results in a Tertiary Hospital.” *Europace* (England) 18, no. 3 (2016): 457–62. <https://doi.org/10.1093/europace/euv106>.

Vats V, DiDomenico RJ, Wojtynek JE, Theobald JC, and Schumock GT. “Hospital Policies for Treatment of Acute Decompensated Heart Failure.” *Ann Pharmacother* (United States) 41, no. 4 (2007): 562–67. <https://doi.org/10.1345/aph.1H664>.

Volz KA, Canham L, Kaplan E, Sanchez LD, Shapiro NI, and Grossman SA. “Identifying Patients with Cellulitis Who Are Likely to Require Inpatient Admission after a Stay in an ED Observation Unit.” *Am J Emerg Med* (United States) 31, no. 2 (2013): 360–64. <https://doi.org/10.1016/j.ajem.2012.09.005>.

Wakai A. “Observation Unit Protocol for Fibrillation.” *Ann Emerg Med* (United States) 41, no. 3 (2003): 421–22; author reply 422. <https://doi.org/10.1067/mem.2003.82>.

Wallins JS, Cajiao KM, McCarthy KJ, Estrada-Roman A, and Gavin MC. “Impact of an Outpatient Cardiology-Managed Urgent Access and Observation Unit on Hospital Admissions.” *Crit Pathw Cardiol* (United States) 18, no. 3 (2019): 113–20. <https://doi.org/10.1097/HPC.0000000000000186>.

Wang H, Watson K, Robinson RD, et al. “Chest Pain Risk Scores Can Reduce Emergent Cardiac Imaging Test Needs With Low Major Adverse Cardiac Events Occurrence in an Emergency Department Observation Unit.” *Crit Pathw Cardiol* (United States) 15, no. 4 (2016): 145–51. <https://doi.org/10.1097/HPC.0000000000000090>.

Wheatley MA, Kapil S, Lewis A, et al. “Management of Minor Traumatic Brain Injury in an ED Observation Unit.” *West J Emerg Med* (United States) 22, no. 4 (2021): 943–50. <https://doi.org/10.5811/westjem.2021.4.50442>.

Wiese M. “Reducing Short-Stay Hospital Admissions by Ruling out Non-ST Elevation Myocardial Infarction and Estimating Coronary Artery Disease Likelihood on an Emergency Department Observation Ward.” *BMJ Qual Improv Rep* (England) 2, no. 1 (2013). <https://doi.org/10.1136/bmjquality.u201080.w695>.

Williams C, Van Ligten MJ, Tomlinson B, et al. “Challenges and Successes in Introducing Coronary CT Angiography in an Emergency Medicine-Run Observation Unit.” *Cureus* (United States) 16, no. 7 (2024): e63620. <https://doi.org/10.7759/cureus.63620>.

Yousuf T, Keshmiri H, Ziffra J, et al. “Impact of Chest Pain Protocol Targeting Intermediate Cardiac Risk Patients in an Observation Unit of an Academic Tertiary Care Center.” *J Clin Med Res* (Canada) 8, no. 2 (2016): 111–15. <https://doi.org/10.14740/jocmr2441w>.

Yun BJ, Borczuk P, Wang L, Dorner S, White BA, and Raja AS. “Evaluation of a Low-Risk Mild Traumatic Brain Injury and Intracranial Hemorrhage Emergency Department Observation Protocol.” *Acad Emerg Med* (United States) 25, no. 7 (2018): 769–75. <https://doi.org/10.1111/acem.13350>.

Zafar MA, Loftus TM, Palmer JP, et al. “COPD Care Bundle in Emergency Department Observation Unit Reduces Emergency Department Revisits.” *Respir Care* (United States) 65, no. 1 (2020): 1–10. <https://doi.org/10.4187/respcare.07088>.

Zucchelli A, Apuzzo R, Paolillo C, et al. “Development and Validation of a Delirium Risk Assessment Tool in Older Patients Admitted to the Emergency Department Observation Unit.” *Aging Clin Exp Res* (Germany) 33, no. 10 (2021): 2753–58. <https://doi.org/10.1007/s40520-021-01792-4>.

Appendix 4: Covidence Data Extraction Tool

Question 1: Country in which the study was conducted

- United States
- Canada
- UK
- Europe (excluding UK)
- Australia
- Asia
- South America
- Other

Question 2: Study Design

- Observational study (cohort, cross-sectional, case-control)
- Non-randomized experimental study
- Randomized controlled trial
- Systematic or scoping review
- Qualitative research
- Case report or series
- Economic evaluation
- Commentary or opinion
- Other

Question 3: Publication Journal

Question 4: Publication Year

Question 5: Patient Population

- All adults (unspecified)
- Geriatrics (65+) specifically
- Non-geriatric adults

Question 6: Type of Emergency Department

- Academic (tertiary or quaternary centers)
- Urban
- Rural
- Community
- Combination
- Unspecified

Question 7: Type of Protocol

- Diagnostic
- Therapeutic
- Both
- Neither
- Unspecified

Question 8: Protocol Specialty

- Addiction Medicine
- Allergy & Immunology
- Cardiology (chest pain, syncope, CHF)
- Cardiac Surgery
- Dermatology
- ENT (oral medicine & dentistry)
- Endocrinology
- Gastroenterology
- Geriatrics
- Gynecology
- Hematology & Oncology
- Infectious Disease
- Nephrology
- Neurology
- Neurosurgery
- Ophthalmology
- Orthopedics
- Pain Management
- Palliative Care
- Plastic Surgery
- Procedure (e.g., colonoscopy, interventional radiology, etc.)
- Psychiatry (anxiety, depression, schizophrenia, suicidal)
- Pulmonology (Respiratory)
- Radiology
- Toxicology (overdose)
- Surgery (General)
- Transplant (Medicine or Surgery)
- Trauma
- Urology
- Vascular Surgery
- Other

Question 9: Protocol Condition

- Abdominal pain
- Allergic reaction
- Atrial fibrillation (or other arrhythmias)
- Back pain
- Cancer-related
- Case Management / Social Interventions (rehab placement, home services & materials, ADLs, limited resources)
- Cellulitis / Wound care
- Chest pain (e.g., acute coronary syndrome, MI, STEMI, angina, revascularization)
- Congestive heart failure
- COPD/Asthma
- Dehydration / Nausea & vomiting
- Dental/ENT management
- Dysglycemia (hyper/hypoglycemia, diabetes, DKA)
- Electrolyte abnormalities (sodium, potassium, magnesium, phosphorus)
- Febrile neutropenia
- Gastroenteritis (nausea, vomiting, diarrhea, abdominal pain)
- General pain control
- GI bleed (upper and lower GI bleed)
- Headache / Migraine
- Head injury / Head bleed
- Musculoskeletal (not including back pain)
- Overdose
- Palliative care/hospice
- Pneumonia
- Psychiatric conditions
- Physical therapy (rehab placement)
- Renal colic / Kidney stones
- Seizure
- Sickle cell pain
- Substance Use (alcohol, tobacco, opioids, drugs)
- Syncope (including near-syncope)
- Telemetry / Vital sign monitoring
- Transient ischemic attack (TIA) / Stroke
- Transfusion
- UTI / Pyelonephritis
- Vertigo / Dizziness
- Viral illness (including COVID)
- Virtual care / Telehealth
- VTE
- Other

Question 10: Outcomes Measured

- Cost ($)
- Hospital Length of Stay (LOS of entire hospitalization)
- Mortality (and Morbidity)
- EDOU Length of Stay (LOS of observation care)
- Patient appropriateness (if the patient placed in EDOU is EDOU-appropriate)
- Patient satisfaction
- Provider adherence
- Provider satisfaction
- Rate of discharge
- Rate of hospitalization
- Resource utilization (# of tests, scans, staff, physical space)
- Return ED visit rate (bouncebacks)
- Throughput (boarding)
- Timeliness (time to intervention, time to test)
- Upgrades to ICU

Appendix 5: Inter-Rater Reliability

Inter-rater agreement for title/abstract and full-text screening was assessed using Cohen’s Kappa statistic. Agreement was modest, reflecting the conceptual ambiguity in distinguishing structured protocols from general observation care descriptions in the literature. To minimize the impact of this variability, all disagreements were resolved through consensus with a senior reviewer.

| **Title & Abstract Review** | |
| --- | --- |
| Reviewers | Cohen’s Kappa |
| HC & MP | 0.667 |

| **Full-Text Review** | |
| --- | --- |
| Reviewers | Cohen’s Kappa |
| HC & MP | 0.352 |

Appendix 6: Distribution of Publication Journals

| **Publication Journal** | **Count** |
| --- | --- |
| Group A | 70 |
| Group B | 24 |
| *Academic Emergency Medicine* | 16 |
| *Western Journal of Emergency Medicine* | 16 |
| *Annals of Emergency Medicine* | 13 |
| *Emergency Medicine Journal* | 10 |
| *Journal of Emergency Medicine* | 9 |
| *Cureus* | 6 |
| *Congestive Heart Failure* | 3 |
| *European Journal of Emergency Medicine* | 3 |

Group A: 1 Publication Each

- *Acute Medicine*
- *Advanced Emergency Nursing Journal*
- *African Journal of Emergency Medicine*
- *Age and Ageing*
- *American Journal of Cardiovascular Disease*
- *American Journal of Therapeutics*
- *Annals of Royal College of Surgeons of England*
- *Antibiotics*
- *ANZ Journal of Surgery*
- *Australasian Journal on Ageing*
- *BJ Psych Open*
- *BMC*
- *BMC Emergency Medicine*
- *BMC Psychiatry*
- *BMJ Quality Improvement Reports*
- *British Medical Journal*
- *Cardiology Clinics*
- *Clinical and Experimental Emergency Medicine*
- *Contemporary Clinical Trials*
- *Current Treatment Options in Cardiovascular Medicine*
- *Disaster Medicine and Public Health Preparedness*
- *Drugs - Real World Outcomes*
- *Endocrine Practice*
- *Epidemiology and Infection*
- *Europace*
- *European Review for Medical and Pharmacological Sciences*
- *IJC Heart & Vasculature*
- *Infectious Diseases and Therapy*
- *Interactive Cardiovascular and Thoracic Surgery*
- *Internal and Emergency Medicine*
- *International Journal of Cardiology Heart & Vasculature*
- *Italian Heart Journal*
- *JEADV*
- *Journal of Accident and Emergency Medicine*
- *Journal of Clinical Gastroenterology*
- *Journal of Clinical Medicine Research*
- *Journal of Knee Surgery*
- *Journal of Medical Toxicology*
- *Journal of Nuclear Cardiology*
- *Journal of Patient Experience*
- *Journal of the American Academy of PAs*
- *Journal of the American Geriatrics Society*
- *Journal of the American Heart Association*
- *Journal of the National Comprehensive Cancer Network*
- *Journal of Trauma*
- *Lung*
- *Managed Care*
- *Medical Care Research and Review*
- *Medicina*
- *Minerva Medica*
- *Neurocritical Care*
- *Nicotine & Tobacco Research*
- *Nursing Economics*
- *Oncology Practice*
- *Pancreatology*
- *Physical Therapy*
- *Pilot and Feasibility Studies*
- *Preventative Cardiology*
- *Psychiatric Services*
- *Respiratory Care*
- *Rev Peru Med Exp Salud Publica*
- *Singapore Medical Journal*
- *Southern Medical Journal*
- *Surgery*
- *The American Journal of Medicine*
- *The Annals of Pharmacotherapy*
- *The Journal of Arthroplasty*
- *The Journal of Family Practice*
- *The Medical Bulletin of Sisli Etfal Hospital*
- *The Neurohospitalist*
- *World Journal of Cardiology*

Group B: 2 Publications Each

- *Aging Clinical and Experimental Research*
- *Cancers*
- *Clinical Cardiology*
- *Clinical Toxicology*
- *International Journal of Emergency Medicine*
- *JACC: Cardiovascular Imaging*
- *Journal of Emergency Nursing*
- *Journal of Hospital Medicine*
- *Journal of Stroke and Cerebrovascular Diseases*
- *Journal of the American College of Emergency Physicians Open*
- *PLOS ONE*
- *The American Journal of Managed Care*
